# Pathology-oriented multiplexing enables integrative disease mapping

**DOI:** 10.1038/s41586-025-09225-2

**Published:** 2025-07-18

**Authors:** Malte Kuehl, Yusuke Okabayashi, Milagros N. Wong, Lukas Gernhold, Gabriele Gut, Nico Kaiser, Maria Schwerk, Stefanie K. Gräfe, Frank Y. Ma, Jovan Tanevski, Philipp S. L. Schäfer, Sam Mezher, Jacobo Sarabia del Castillo, Thiago Goldbeck-Strieder, Olga Zolotareva, Michael Hartung, Fernando M. Delgado Chaves, Lukas Klinkert, Ann-Christin Gnirck, Marc Spehr, David Fleck, Mehdi Joodaki, Victor Parra, Mina Shaigan, Martin Diebold, Marco Prinz, Jennifer Kranz, Johan M. Kux, Fabian Braun, Oliver Kretz, Hui Wu, Florian Grahammer, Sven Heins, Marina Zimmermann, Fabian Haas, Dominik Kylies, Nicola Wanner, Jan Czogalla, Bernhard Dumoulin, Nikolay Zolotarev, Maja Lindenmeyer, Pall Karlson, Jens R. Nyengaard, Marcial Sebode, Sören Weidemann, Thorsten Wiech, Hermann-Josef Groene, Nicola M. Tomas, Catherine Meyer-Schwesinger, Christoph Kuppe, Rafael Kramann, Alexandre Karras, Patrick Bruneval, Pierre-Louis Tharaux, Diego Pastene, Benito Yard, Jennifer A. Schaub, Phillip J. McCown, Laura Pyle, Ye Ji Choi, Takashi Yokoo, Jan Baumbach, Pablo J. Sáez, Ivan Costa, Jan-Eric Turner, Jeffrey B. Hodgin, Julio Saez-Rodriguez, Tobias B. Huber, Petter Bjornstad, Matthias Kretzler, Olivia Lenoir, David J. Nikolic-Paterson, Lucas Pelkmans, Stefan Bonn, Victor G. Puelles

**Affiliations:** 1 Department of Clinical Medicine, Aarhus University, Aarhus, Denmark; 2 Department of Pathology, Aarhus University Hospital, Aarhus, Denmark; 3 Institute of Medical Systems Bioinformatics, Center for Biomedical AI (bAIome), Center for Molecular Neurobiology Hamburg (ZMNH), University Medical Center Hamburg-Eppendorf, Hamburg, Germany; 4 III. Department of Medicine, University Medical Center Hamburg-Eppendorf, Hamburg, Germany; 5 Hamburg Center for Kidney Health (HCKH), University Medical Center Hamburg-Eppendorf, Hamburg, Germany; 6 Division of Nephrology and Hypertension, Department of Internal Medicine, The Jikei University School of Medicine, Tokyo, Japan; 7 Department of Molecular Life Sciences, University of Zurich, Zurich, Switzerland; 8 Department of Medical Oncology and Hematology, University Hospital Zurich and University of Zurich, Zurich, Switzerland; 9 Department of Nephrology, Monash Medical Centre and Department of Medicine, Monash University, Melbourne, Victoria Australia; 10 Institute for Computational Biomedicine, Heidelberg University and Heidelberg University Hospital, Heidelberg, Germany; 11 Translational Spatial Profiling Center, Heidelberg University Hospital, Heidelberg, Germany; 12 Institute for Computational Systems Biology, University of Hamburg, Hamburg, Germany; 13 Data Science in Systems Biology, TUM School of Life Sciences, Technical University of Munich, Freising, Germany; 14 Euroimmun, Lübeck, Germany; 15 Department of Chemosensation, Institute for Biology II, RWTH Aachen University, Aachen, Germany; 16 Institute for Computational Genomics, RWTH Aachen University, Aachen, Germany; 17 Faculty of Medicine, Institute of Neuropathology, and Signalling Research Centres BIOSS and CIBSS, University of Freiburg, Freiburg, Germany; 18 Department of Urology and Pediatric Urology, University Hospital RWTH Aachen, Aachen, Germany; 19 Department of Urology and Kidney Transplantation, Martin-Luther-University, Halle, Germany; 20 Cell Communication and Migration Laboratory, Institute of Biochemistry and Molecular Cell Biology, Center for Experimental Medicine, University Medical Center Hamburg-Eppendorf, Hamburg, Germany; 21 Martin Zeitz Center for Rare Diseases, University Medical Center Hamburg-Eppendorf, Hamburg, Germany; 22 Core Center for Molecular Morphology, Section for Stereology and Microscopy, Aarhus University, Aarhus, Denmark; 23 Danish Pain Research Center, Department of Clinical Medicine, Aarhus University, Aarhus, Denmark; 24 I. Department of Medicine, University Medical Center Hamburg-Eppendorf, Hamburg, Germany; 25 Department of Pathology, University Medical Centre Hamburg-Eppendorf, Hamburg, Germany; 26 Institute of Pathology, Nephropathology Section, University Medical Center Hamburg-Eppendorf, Hamburg, Germany; 27 Institute of Pharmacology, University of Marburg, Marburg, Germany; 28 Center for Experimental Medicine, Institute of Cellular and Integrative Physiology, University Medical Center Hamburg-Eppendorf, Hamburg, Germany; 29 Department of Nephrology, RWTH Aachen University, Medical Faculty, Aachen, Germany; 30 Department of Nephrology, Georges Pompidou European Hospital, APHP, Université Paris Cité, Paris, France; 31 Université Paris Cité, Inserm, Paris Cardiovascular Research Center (PARCC), Paris, France; 32 V. Department of Medicine, University Medical Center Mannheim, University of Heidelberg, Mannheim, Germany; 33 Department of Internal Medicine, Division of Nephrology, University of Michigan, Ann Arbor, MI USA; 34 Department of Pediatrics, Division of Endocrinology and Department of Medicine, Division of Metabolism, Endocrinology and Nutrition, University of Washington, Seattle, WA USA; 35 Department of Mathematics and Computer Science, University of Southern Denmark, Odense, Denmark; 36 Department of Pathology, University of Michigan, Ann Arbor, MI USA

**Keywords:** Translational research, Kidney diseases

## Abstract

The expression and location of proteins in tissues represent key determinants of health and disease. Although recent advances in multiplexed imaging have expanded the number of spatially accessible proteins^1–3^, the integration of biological layers (that is, cell structure, subcellular domains and signalling activity) remains challenging. This is due to limitations in the compositions of antibody panels and image resolution, which together restrict the scope of image analysis. Here we present pathology-oriented multiplexing (PathoPlex), a scalable, quality-controlled and interpretable framework. It combines highly multiplexed imaging at subcellular resolution with a software package to extract and interpret protein co-expression patterns (clusters) across biological layers. PathoPlex was optimized to map more than 140 commercial antibodies at 80 nm per pixel across 95 iterative imaging cycles and provides pragmatic solutions to enable the simultaneous processing of at least 40 archival biopsy specimens. In a proof-of-concept experiment, we identified epithelial JUN activity as a key switch in immune-mediated kidney disease, thereby demonstrating that clusters can capture relevant pathological features. PathoPlex was then used to analyse human diabetic kidney disease. The framework linked patient-level clusters to organ disfunction and identified disease traits with therapeutic potential (that is, calcium-mediated tubular stress). Finally, PathoPlex was used to reveal renal stress-related clusters in individuals with type 2 diabetes without histological kidney disease. Moreover, tissue-based readouts were generated to assess responses to inhibitors of the glucose cotransporter SGLT2. In summary, PathoPlex paves the way towards democratizing multiplexed imaging and establishing integrative image analysis tools in complex tissues to support the development of next-generation pathology atlases.

## Abstract (web summary)

Pathology-oriented multiplexing (PathoPlex) represents a framework for widespread access to multiplexed imaging and computational image analysis of clinical specimens at a relatively high throughput and subcellular resolution.

## Main

Spatial biology technologies have gained increased attention recently as they provide molecular insights into transcriptomic and proteomic expression while preserving histological context^1^. The term multiplexed imaging refers to the expansion of antibody-based labelling beyond conventional limits (that is, 3–4 antibodies per section)^2,3^. Multiple commercial systems are available with varying performance and cost. For example, methods based on mass spectrometry^4,5^ require specialized equipment and antibody conjugation to metals, enabling spatial projections with high precision and reproducibility at cellular resolution (between 250 and 1,000 nm per pixel). Alternatively, microscopy-based methods^6,7^ are more economically accessible and rely on the cyclic detection of DNA-conjugated antibody panels or direct immunofluorescence using fixed integrated widefield microscopy. Although such methods achieve an image resolution of 200–300 nm per pixel, there is a trade-off between detection speed and signal amplification. Results from studies that used both mass spectrometry and microscopy-based methods^8,9^ aligned well with comprehensive reviews of the literature^10^ that reported panels ranging between 30 and 60 antibodies. This body of work set the foundation for the development of image analysis strategies that focused on the identification of cell identities and states through cell segmentation^11–14^.

In 2018, iterative indirect immunofluorescence imaging (4i)^15^ was introduced as an open-source tool for multiplexed imaging and advanced image analysis. These techniques were based on the use of unmodified commercial antibodies in cyclic rounds of immunofluorescence imaging through simple steps of chemical elution and flexible light microscopy. 4i was originally applied in vitro using 41 antibodies at a resolution of 165 nm per pixel, which enabled the detection of functional multilayered subcellular features of cell injury through pixel-level analysis. To our knowledge, there is only one study that recreated the original 4i protocol in multicellular specimens^16^ with sufficient multiplexed imaging depth (21 imaging cycles for 54 markers) and image resolution (160 nm per pixel) to perform pixel-based image analysis. However, despite being one of the largest and most complex datasets available, the outputs derived from multiplexed imaging have primarily been used to recapitulate known cellular events during organ development. In this context, we postulate that the potential of multiplexed imaging methods to define tissue-based integrative features associated with health and disease remains underexplored.

## Current state-of-the-art

A study^10^ that discussed the current landscape of antibody-based multiplexed imaging showed that there is a diverse range in performance among the methods. From all the different criteria that can be used to define the advantages and limitations of each method, we propose two criteria to evaluate the potential to support image analysis tools that aim to integrate multiple biological layers (Supplementary Fig. 1a): the number of markers (panel size) and the image resolution per pixel. Although it is evident that panel size directly affects the scope of processes that can be analysed, image resolution and the biological insights gained from it are harder to appreciate. To illustrate the importance of image resolution, we compared a mass-spectrometry-based method (Supplementary Fig. 1b) and a microscopy-based method (Supplementary Fig. 1c) for analysing kidney samples using markers of cell identity and DNA. This comparison highlighted an obvious resolution mismatch that had a clear impact on the ability to delineate subcellular structures (for example, nuclei and even nucleoli) and the borders of neighbouring cells (for example, renal endothelial and epithelial cells).

Among the reported multiplexing methods^10^, the average panel size is approximately 37 markers with an average resolution of 267 nm per pixel. The most used systems, such as imaging mass cytometry (IMC; 40 markers at 1,000 nm per pixel) and co-detection by indexing (CODEX; 56 markers at 250 nm per pixel), provide reliable references of current commercial standards. Thus, it is not surprising that most studies in the field of antibody-based spatial proteomics fundamentally rely on single-cell segmentation as a core step, similar to the approaches used in spatial transcriptomics^17,18^. That is, neither the resolution nor the panel size provide the foundation for more integrative image analysis. Furthermore, most studies of organs that have high cell density (for example, the kidney) typically report cell identity and state^19,20^ but do not provide integrative data across biological domains. These limitations represent an opportunity for the next generation of multiplexed imaging methods to scale panel sizes beyond current limits. Moreover, computational tools can be built to extract hallmarks of health and disease by weighting and connecting the contributions of each biological layer (Supplementary Fig. 2).

## Towards next-generation multiplexed imaging

**Fig. 1:**
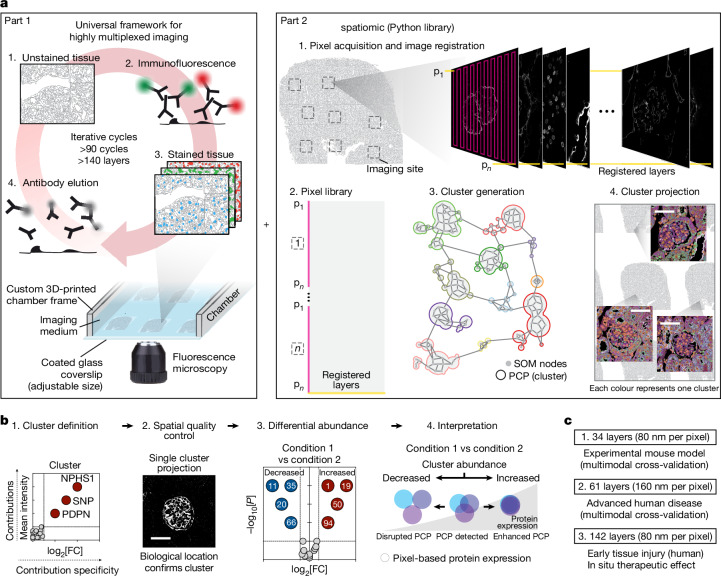
PathoPlex. **a**, PathoPlex represents a combination between a universal framework for highly multiplexed imaging in pathological tissues (left) and a Python library (spatiomic) to analyse protein co-expression patterns (PCPs) or clusters (right). **b**, Step-by-step interpretation of generated clusters. **c**, Summary of all experimental datasets in this study. Scale bars, 50 μm. FC, fold change; p, pixel.

Here we introduce PathoPlex, a scalable, quality-controlled and interpretable framework. It combines highly multiplexed imaging at subcellular resolution with an open-source software package to facilitate integrative analyses of formalin-fixed paraffin-embedded (FFPE) specimens (Fig. 1a).

In brief, multiplexed imaging is performed in iterative cycles, whereby indirect immunofluorescence labelling is conducted first, followed by image acquisition by fluorescence microscopy (for example, widefield or confocal) and subsequent antibody elution (Fig. 1a, part 1). To prevent tissue lifting, we recommend coating the glass surfaces with poly-d-lysine for small-scale experiments or with (3-aminopropyl)triethoxysilane (APTES) for large-scale experiments, as APTES is more efficient at preventing tissue detachment compared with poly-d-lysine (Methods). In this report, our largest experiment included 95 imaging cycles with antibodies against 150 proteins and 20 quality-control imaging cycles with only secondary antibodies for a total of 170 layers. After detailed examination, we included 142 (122 protein and 20 quality control) layers for analyses, which generated >600 billion available pixels. It is worth noting that the tissues remained stable and did not show signs of damage within 95 imaging cycles, which suggests that this is not the limit of the technology.

To accommodate the scale of these datasets and to enable modular composition and extendibility of bioinformatic analyses, we developed a high-performance computing library for spatial proteomics (which we term spatiomic) that leverages various algorithms based on graphics processing units (GPUs)^21,22^, integrates common data formats^23^ and is freely available as a Python package through the PyPi registry (Fig. 1a, part 2). The package spatiomic features multiple registration algorithms to align images of individual markers for joint analyses. To identify protein co-expression patterns, spatiomic includes modules to preprocess images, obtain a representative subsample, reduce dimensionality using self-organizing maps (SOMs), construct a similarity-based neighbourhood graph and perform graph clustering^24^. Co-expression patterns can be consistently identified across all images of an experimental dataset and spatially projected. As these co-expression patterns are generated on the basis of pixel-level clustering, from now on, we refer to them as ‘clusters’.

Each cluster has the potential to represent a biological process and warrants further interpretation (Fig. 1b). As a first step, the individual contribution of each marker to the cluster was analysed to define the specific co-expression pattern that each cluster represents. For this reason, the mean normalized intensity (the level of contribution per marker) and the log_2_-transformed fold change in relation to the mean of other clusters (the specific contribution of each marker) were systematically evaluated. As each marker represents proteins with known or predicted locations, distributions and expression patterns, it can be projected back into space for visual validation. Cluster abundance was used as a quantifiable metric to statistically compare conditions and to isolate differentially expressed clusters. Notably, changes in cluster abundance can result not only from differences in protein expression levels but also from changes in protein distribution (for example, cytoplasmic to nuclear shifts).

As an overview, we first provided proof-of-principle and quality-control datasets in three different organs (<30 markers at a resolution of 160 nm per pixel). PathoPlex was then validated using the kidney as a model organ with high cellular density and structural complexity through in-depth analyses of three additional datasets (Fig. 1c). These datasets were obtained from the following sources: (1) an experimental mouse model of immune-mediated kidney disease (34 markers at 80 nm per pixel); (2) clinical biopsy samples from individuals diagnosed with advanced diabetic kidney disease (DKD) (61 markers at 160 nm per pixel); and (3) research biopsy samples from individuals diagnosed with youth-onset type 2 diabetes (T2D) (142 markers at 80 nm per pixel) without pathological signs of DKD, including a subset of individuals with short-term treatment with SGLT2 inhibitors.

## Proof-of-principle and quality controls

Proof-of-principle experiments were performed on the basis of representative samples from autoimmune hepatitis, meningioma and focal segmental glomerulosclerosis (Supplementary Fig. 3) and controls in human liver, brain and kidney, respectively (Supplementary Fig. 4) showing broad applicability in pathology and a wide potential for marker selection, including transcription factors, enzymes, structural proteins, subcellular domains, cell surface receptors and phosphorylation targets.

**Extended Data Fig. 1:**
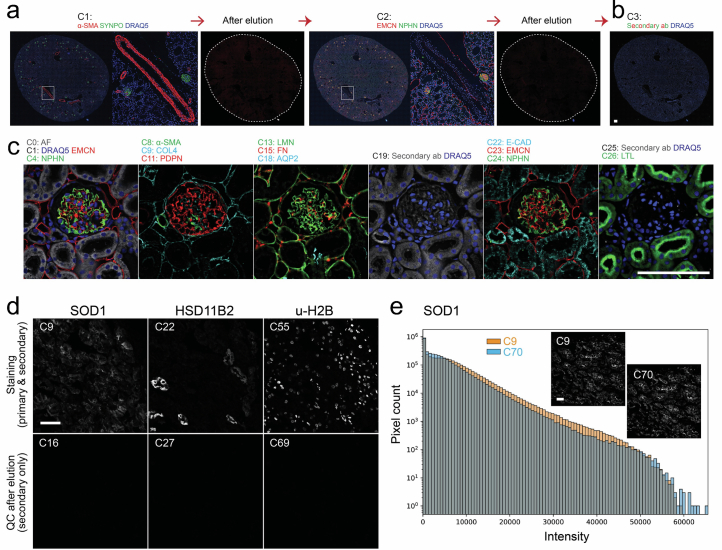
Practical quality control steps. (a) Full elution of antibodies and (b) absence of remnant signals when re-staining with secondary antibodies. (c) Specificity of staining and constancy of re-stained structures over multiple imaging cycles. (d) Secondary-only cycles do not show signal amplification. (e) Re-staining of SOD1 after 61 cycles, showing very strong agreement between intensity distributions. vWF, von Willebrand factor; SRB1, Scavenger receptor class B member 1; PDI, Protein disulfide isomerase; EZR, Ezrin; p-rp-S6, phospho-ribosomal protein S6; p-H3, phospho-histone-H3; PCNA, Proliferating cell nuclear antigen; COL4, Collagen type IV; LAMP1, lysosomal-associated membrane protein 1;VMT, Vimentin; FN, Fibronectin; WT1, Wilms tumor 1; ACE2, Angiotensin-converting enzyme 2; CK19, Cytokeratin 19; ANXA3, Annexin 3; LTL, Lotus tetragonolobus lectin; α-SMA, alpha-smooth muscle actin (ACTA2); SYNPO, Synaptopodin; EMCN, Endomucin; NPHN, Nephrin; ab, antibody; AF, autofluorescence; PDPN, Podoplanin; LMN, Laminin; AQP2, Aquaporin 2; E-CAD, E-cadherin; SOD1: Superoxide Dismutase 1; HSD11B2: 11-β-hydroxysteroid dehydrogenase type 2; u-H2B: ubiquitylated histone 2B. Scale bars represent 100 µm.

Quality-control criteria for PathoPlex were first established in murine tissues and then extended to human specimens. In brief, consecutive imaging cycles of an antibody panel constituted the first level of control. This step was important because incomplete elution might lead to cross-reactivity with subsequent cycles or residual signals from the previous cycle. The second level of control involved direct imaging after elution to confirm the lack of fluorescent signals (Extended Data Fig. 1a). The third level of control included imaging cycles using secondary antibodies without previous incubation of primary antibodies (secondary-only cycles). This step ensured the absence of remnant viable primary antibodies and generated additional layers that could be included in image analyses (Extended Data Fig. 1b). The fourth level of control involved successful re-staining after multiple imaging cycles (Extended Data Fig. 1c). This stage was used to confirm that the epitope is preserved and the effectiveness of antibody elution. Furthermore, we applied practical quality-control steps for human tissue samples throughout 95 imaging cycles. This strategy showed complete elution efficiency using secondary-only cycles (Extended Data Fig. 1d and Supplementary Figs. 5 and  6) and effective re-stainings after 60 cycles (Extended Data Fig. 1e and Supplementary Fig. 7).

Once all the imaging cycles were completed, image alignment was performed to account for potential shifts during the various cycles. It is well established that nuclei can be easily stained, but commonly used labels are either unstable (for example, 4′,6-diamidino-2-phenylindole (DAPI)) or expensive (for example, DRAQ5). For this reason, we introduce *N*-hydroxysuccinimide ester (NHS-E), a pan-protein label commonly used in super-resolution microscopy^25^. NHS-E consistently generated reference images for alignment and showed equally high performance compared with nuclear references (Supplementary Fig. 8). Moreover, NHS-E can be used to segment tissue-containing areas to limit the analysis of regions with potential nonspecific binding. Unlike DAPI or DRAQ5, which need constant re-staining every imaging cycle, NHS-E requires a single application at the beginning of the protocol and remains stable for up to 95 cycles.

## Practical considerations

**Extended Data Fig. 2:**
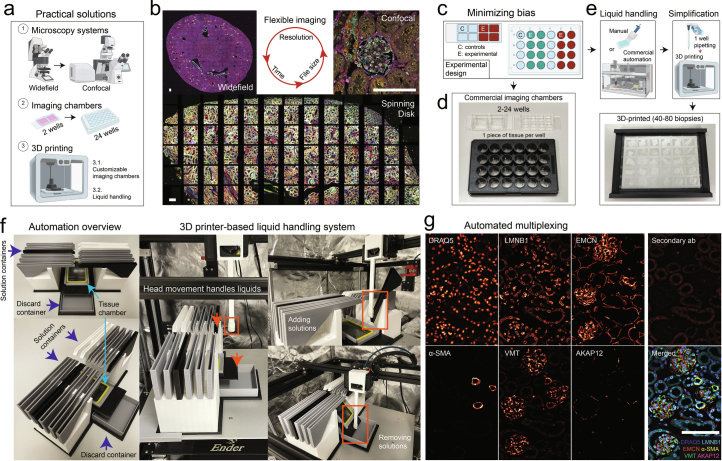
Practical solutions to minimize bias introduction. (a) Schematic overview of all strategies deployed. (b) PathoPlex is compatible with multiple microscopy systems, including widefield, spinning disk and laser confocal, which directly determine imaging time, file size and image resolution. (c) Strategies to minimize bias introduction are based on creating self-contained batches, where tissues are processed in parallel using imaging chambers that contain specimens representing all experimental groups, including controls. (d) Suggested imaging chambers for histopathological studies (2–24 wells). (e) Multi-well pipetting can be performed manually or using liquid handling systems. Alternatively, 3D printing can be used to customize a 1-well imaging chamber, which simplifies liquid handling. (f) In addition, a 3D printer can also be used as a liquid handling system. The printer head is used to automate liquid addition and removal. (g) Specificity, and elution controls of 3D printer-based imaging cycles. LMNB1, Lamin B1 (cycle 1); EMCN, Endomucin (cycle 1); α-SMA, alpha-smooth muscle actin or ACTA2 (cycle 2); VMT, Vimentin (cycle 4); AKAP12, A-Kinase Anchoring Protein 12 (cycle 4); Secondary ab (QC cycle; cycle 6). Scale bars represent 100 µm. Parts of panels a, c and e were created using BioRender (https://biorender.com).

PathoPlex combines different strategies to optimize performance and to minimize the potential introduction of batch effects, including adaptable microscopy, accessible and customizable imaging set-ups and low-cost automatization of liquid handling (Extended Data Fig. 2a). PathoPlex can be implemented using any inverted system for fluorescence microscopy, including widefield, spinning disk and confocal, which provides flexibility in terms of image resolution, scanning time and file size (Extended Data Fig. 2b).

It is worth mentioning that classical pathology protocols and some multiplexing technologies may inadvertently introduce batch effects, as specimens are processed as individual slides. By contrast, PathoPlex uses imaging chambers that enable the parallel processing of multiple tissues in single runs. Each imaging chamber is organized as an independent and self-contained experiment by including both control and experimental samples (Extended Data Fig. 2c). Considering the size of average unmodified histopathological samples, commercial solutions can be used to process between 2 and 24 intact samples at the same time (Extended Data Fig. 2d). However, as the number of wells increases, manual pipetting increases the likelihood of user error. Although this source of error can be mitigated through automation, commercially available liquid-handling systems are often expensive and not accessible to the wider scientific community. For this reason, PathoPlex introduces two practical 3D printing-based strategies to simplify liquid handling. The first approach involved the creation of a large unified single-well imaging chamber (11 × 7.4 cm) using a 3D-printed frame (Extended Data Fig. 2e and Supplementary Fig. 9a) that can hold 40 intact human kidney biopsy samples (approximately 100 mm^2^ in size) and even higher numbers of smaller biopsy samples (for example, with size extrapolation, this equates to approximately 77 skin biopsy samples). The second strategy involved the automation of staining and elution cycles. To achieve this, we repurposed a 3D printer as a low-cost liquid handling system, with the printer head controlling liquid addition and removal (Extended Data Fig. 2f, Supplementary Fig. 9b and Supplementary Video 1). This approach produced successful staining and elution cycles (Extended Data Fig. 2g), saving approximately 70% hands-on time with minimal user input (Supplementary Fig. 9c). Although an automated solution for multiplexed imaging using 4i principles has been previously reported^26^, our universal framework provides users with the flexibility to design their experiment according to their needs, including sample size and image resolution.

## Proof-of-concept in experimental disease

**Fig. 2:**
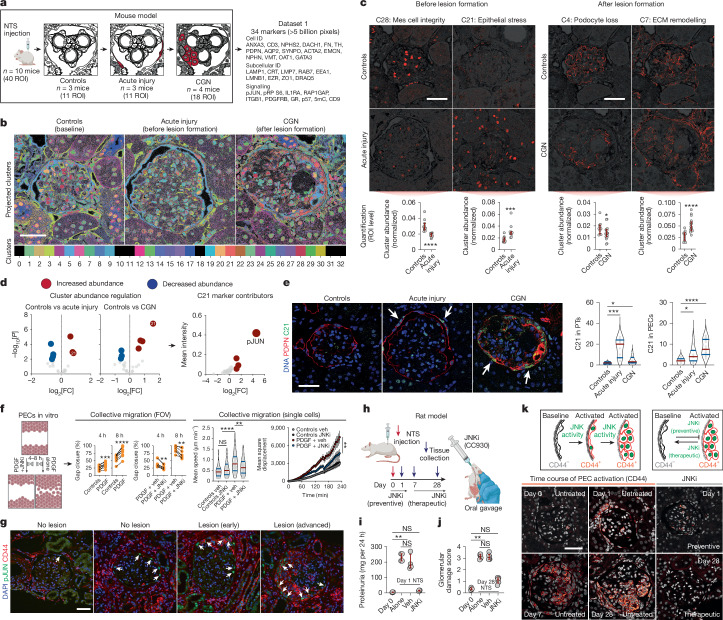
Identification of epithelial JUN activity as a key switch in immune-mediated kidney disease. **a**, Schematic overview for the proof-of-concept experiment in a mouse model of immune-mediated kidney disease before (acute injury) and after (CGN) pathological lesion formation (*n* = 10 mice; ROIs = 40). NTS, nephrotoxic serum; details of the antibody panel are provided in Supplementary Table 1. **b**, Spatiotemporal distribution of colour-coded clusters. **c**, Examples of interpretable clusters (C28, C21, C4 and C7) of biological significance. Each dot represents an ROI, which was used as an independent observation (*n* = 11 ROIs for controls, *n* = 11 ROIs for acute injury and *n* = 18 ROIs for CGN) and red bars represent medians and inter-quartile ranges. Mes, mesangial. **d**, Identification of C21 (with pJUN as a top contributor) as a key regulated pathomechanism before and after lesion formation. **e**, Images of the spatiotemporal distribution of C21 (left) and cell-specific frequency (right) among tubular epithelial cells and PECs. **f**, Treatment with a JNK inhibitor (JNKi) reduces the PDGF-mediated collective migration of murine PECs in vitro. In ‘collective migration’, error bars represent upper and lower limits. Data are from four biological replicates. Veh, vehicle. **g**, Confirmation of pJUN expression in PECs during different lesion stages among human kidney biopsy samples (*n* = 12 patients and *n* = 3 healthy individuals), which was also associated with CD44 co-expression. **h**, Schematic overview of the use of a JNKi as a preventive strategy (before NTS) and a therapeutic strategy (7 days after NTS) during the progression of immune-mediated kidney disease in a rat model of CGN. **i**,**j**, Proteinuria (*n* = 4 rats for all groups) and glomerular damage (*n* = 4 rats for day 0, *n* = 6 rats for all other groups; red bars represent medians and interquartile ranges) show a direct preventive (**i**) and therapeutic (**j**) effect of the JNKi. **k**, Using expression of CD44 as a readout of PEC activation, we confirmed the effect of the JNKi on PEC activation (using all rats available from **i** and **j**). Differential cluster abundance analysis used a two-sided *t*-test. Cluster composition analysis relied on a two-sided *t*-test with Holm–Šidák correction. For other comparisons, two-sided Mann–Whitney, Kruskal–Wallis with Dunn, analysis of variance (ANOVA) with Dunnett T3 or ANOVA with Holm–Šidák tests were used depending on the number of comparisons. ****P < 0.0001, ***P < 0.001, **P < 0.01, *P < 0.05 or not significant (NS). Scale bars, 50 µm (**c**,**e**,**g**,**k**). Diagrams in **a**, **f** and **h** were created using BioRender (https://biorender.com). Source Data

Next, we performed a proof-of-concept experiment, whereby PathoPlex was used to analyse the pathophysiology of a well-characterized mouse model of immune-mediated kidney disease^27^. These mice exhibit a clear disease course that ranges from acute injury to crescentic glomerulonephritis (CGN). That is, protein loss in the urine (proteinuria), the subsequent development of pathological lesions (crescents) in the renal-filtering units (glomeruli) and progressive loss of kidney function. A total of 34 markers were used at a resolution of 80 nm per pixel to acquire approximately 5 billion pixels in 40 regions of interest (ROIs) centred on individual glomeruli (Fig. 2a). The antibody panel was designed to detect cell identities, subcellular compartments and signalling pathway activity (Supplementary Table 1). From a total of 33 generated clusters, 27 clusters were biologically defined (Supplementary Table 2). Significant changes in cluster abundance during the disease course (Fig. 2b) were used as a metric to define integrative features (Fig. 2c) that reflected well-characterized pathogenic processes^28–31^.

**Extended Data Fig. 3:**
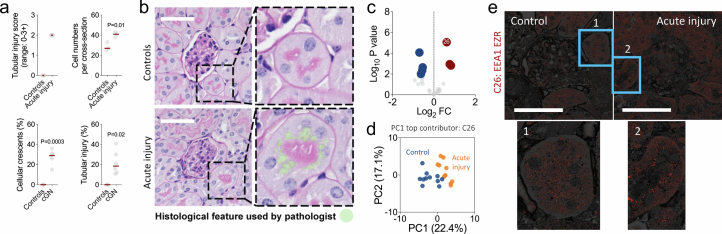
PathoPlex complements pathology evaluations. 2 expert pathologists analyzed all samples and defined all 3 groups with 100% accuracy. (a) Quantitative pathology analyses to differentiate acute injury and CGN vs controls, showing compartment-specific changes in acute injury and CGN. Each dot represents one mouse (N = 3 controls, N = 3 acute injury, and N = 6 CGN; red line represents medians). Two-sided t tests and Mann Whitney tests were used. (b) While mice with CGN have overt lesions, those with acute injury show only minor abnormalities, namely subtle vacuolation in the proximal tubuli (green). (c) Volcano plot showing all regulated clusters comparing controls to acute injury, featured cluster 26 (C26). (d) Principal component (PC) analysis revealed clear separation between images from controls (blue) and acute injury (orange) specimens with C26 being the top contributor to PC1. (e) Spatial projections of cluster 26 (C26) show a regulation pattern in the luminal side of kidney tubuli, a similar location to the one used for pathologists to define acute injury. ****P < 0.0001, ***P < 0.001; EEA1: Early Endosome Antigen 1, EZR: Ezrin. Scale bars represent 60 µm.

Additional histopathological sections from the same experimental animals were carefully evaluated by two expert renal pathologists in a blinded fashion, who were specifically asked to diagnose and stage disease and to quantify structural changes (Extended Data Fig. 3a). Images of CGN were defined by significant vascular injury and the presence of crescentic lesions, whereas acute injury was determined by the vacuolation of tubular cells (Extended Data Fig. 3b). In line with these findings, cluster 26 (which included contributions from early endosome antigen 1 and ezrin) was more abundant in disease than in control samples (Extended Data Fig. 3c), a finding that represented a characteristic feature of acute disease (Extended Data Fig. 3d). Notably, the spatial distribution of cluster 26 corresponded to the location and pattern used by expert pathologists for diagnosis and staging (Extended Data Fig. 3e). This result suggests that PathoPlex can detect pathogenesis-related tissue alterations— similar to those used by human experts—in an unsupervised manner.

## Identification of pathway activity

Previous studies have shown that modulation of JNK signalling can lead to substantial protective effects in kidney autoimmunity and fibrosis^32,33^. The proposed cellular targets of JNK inhibitors have mostly been immune cells (that is, activated macrophages). However, effector cells of crescent formation are parietal epithelial cells (PECs). During crescent formation, these cells are activated through processes that are mediated by growth factors (for example, platelet-derived growth factor (PDGF))^34^, and their increased potential for proliferation and migration is regulated through the de novo expression of the glycoprotein CD44 (ref. ^35^) and the tetraspanin CD9 (ref. ^36^).

**Extended Data Fig. 4:**
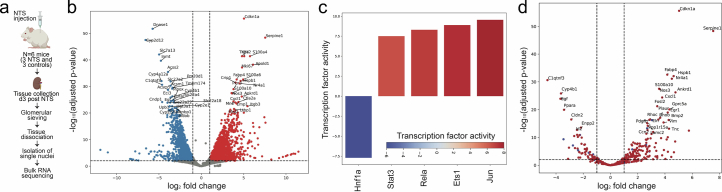
Transcriptional profiling in experimental crescentic nephritis identifies JUN as a top regulated gene. (a) Schematic description of experimental design with nephrotoxic serum (NTS) glomerulonephritis and glomerular nuclear isolation for bulk RNA sequencing. (b) Volcano plots show transcriptionally regulated genes at day 3 post NTS injection. (c) Transcription factor activity score, and (d) differential expression of JUN-regulated targets, where blue means downregulated by JUN and red means upregulated by JUN. Differential gene expression analysis was performed using *PyDESeq2* combining single-factor analysis using Wald tests with log_2_ fold-change shrinkage using approximate posterior estimation generalized linear models. Parts of panel a were created using BioRender (https://biorender.com).

We performed bulk RNA sequencing on nuclei isolated from mice with immune-mediated kidney disease to clarify this issue (Extended Data Fig. 4a). Analyses of the differentially expressed genes (Extended Data Fig. 4b) identified the transcription factor JUN with the highest activity score (Extended Data Fig. 4c), as calculated from the differential expression of JUN-regulated targets (Extended Data Fig. 4d). Notably, JUN has a crucial role in AP-1 activation through JNK, which mediates CD44 signalling^37^. As a readout of JUN activity, its phosphorylated protein product JUN(Ser63) (pJUN) was included in our antibody panel. Cluster 21 featured pJUN as a top contributor and was consistently increased in both acute and CGN disease states compared with controls (Fig. 2d). Cluster 21 was essentially restricted to PECs and tubular cells, with a high frequency in tubular cells during acute injury and a gradual increase in PECs during disease progression to CGN (Fig. 2e). As tubular cells do not represent an effector population during crescent formation, we turned our full attention to the role of JUN activity in PECs.

## Multimodal cross-species validation

**Extended Data Fig. 5:**
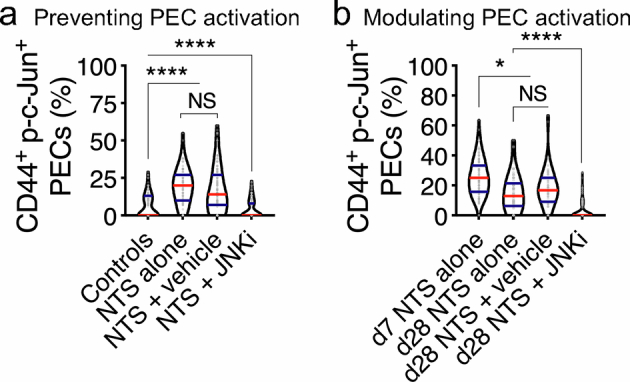
c-Jun activity in a rat model of crescentic nephritis. (a) Percentage of CD44+phospho-c-Jun+ parietal epithelial cells (PECs) among controls, NTS injected day 1 (alone and vehicle) and NTS injected day 1 with a preventive JNK inhibitor (JNKi) administration. (b) Percentage of CD44+phospho-c-Jun+ PECs among NTS injected day 7 (alone), NTS injected d28 (alone and vehicle) and NTS injected day 28 with JNKi administration started at day 7 post NTS injection.

As an initial validation step, we evaluated the effect of JUN activity modulation in PECs. To this end, PEC activation (that is, increased migration) was induced in vitro using PDGF^36^. PEC migration was attenuated with the JNK inhibitor (JNKi) CC930 (also known as tanzisertib) in two independent experimental set-ups. Results from both of these experiments confirmed that CC930 has a direct effect on activated murine PECs (Fig. 2f). In a second validation step, we analysed human biopsy samples from patients diagnosed with CGN to delineate JUN activity during the progression of human crescentic lesions (*n* = 12 patients and *n* = 3 healthy participants). Normal glomeruli from healthy individuals and from individuals with CGN showed that pJUN was expressed in scattered PECs without CD44 expression. As pathological lesions in CGN develop in a focal pattern, some glomeruli appeared normal and only a subset exhibited crescent characteristics, all in the same patient sample. Although some glomeruli showed abundant pJUN^+^CD44^–^ PECs, pJUN^+^CD44^+^ PECs were exclusively found in CGN samples (Fig. 2g), which indicated an association between JUN activity and PEC activation in human specimens. In a third validation step, CGN was modelled in rats to test the efficacy of CC930 as a preventive strategy (before disease induction) or as a therapeutic strategy initiated 7 days after disease induction (Fig. 2h). Proteinuria was substantially decreased in the preventative study (Fig. 2i) and glomerular damage was mitigated with interventional treatment (Fig. 2j), which included substantial modulation of CD44 expression in PECs (Fig. 2k and Extended Data Fig. 5). Together, these data confirmed that PathoPlex-derived clusters can identify actionable pathological features with high spatial precision.

## Integrative mapping of human disease

**Fig. 3:**
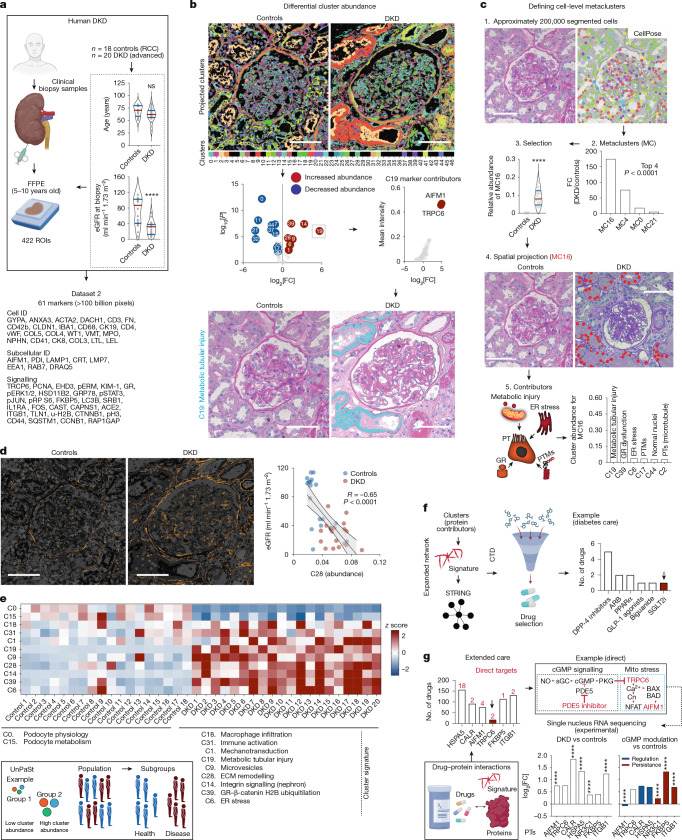
PathoPlex as a tool to analyse human DKD. **a**, Schematic overview of the experimental design to compare control and DKD specimens (*n* = 38 18 controls, 20 DKD; ROIs = 422). Details of the antibody panel are provided in Supplementary Table 3. RCC, renal cell carcinoma. **b**, Scheme of cluster identification to differential abundance and cluster definition. We show the example of C19, which represents metabolic tubular injury (with TRPC6 and AIFM1 as top contributors). **c**, Single-cell segmentation reveals disease-specific cell-level metaclusters (MCs). PTs, proximal tubules; PTMs, post-translational modifications. **d**, Mean cluster abundances correlate with patient-level renal function (linear regression with 95% confidence interval). For this example, cluster 28 represents ECM remodelling and is inversely associated with estimated glomerular filtration rate (eGFR). **e**, Unsupervised bicluster analysis for patient stratification differentiates between DKD and control specimens with perfect accuracy and isolates a subset of biologically meaningful clusters. **f**, Druggabilty profiling of standard care. The top contributors of clusters selected in **b** were used as a DKD signature that was extended using open-access tools (that is, STRING), and then cross-referenced to the CTD to select a subset of drugs. Multiple medications for the standard care of diabetes interacted with our expanded DKD signature. **g**, Drug–protein interactions were quantified for our DKD signature. One example is PDE5 inhibitors as potential modulators of TRPC6–AIFM1 through cGMP signalling, which was confirmed through a re-analysis of public single-nucleus RNA-sequencing data^47^. Differential cluster abundance analysis used a two-sided *t*-test with Benjamini–Hochberg correction. Cluster composition analysis relied on a two-sided *t*-test with Holm–Šidák correction. Correlation analysis was performed using two-sided Spearman’s rank coefficient. For other comparisons, two-sided *t*-test, Mann–Whitney or Kruskal–Wallis tests were used depending on the number of comparisons. ****P < 0.0001, ***P < 0.001, **P < 0.01, *P < 0.05. Scale bars, 100 µm (**b**–**d**). Diagrams in **a**,**e**,**f** and **g** were created using BioRender (https://biorender.com). Source Data

**Extended Data Fig. 6:**
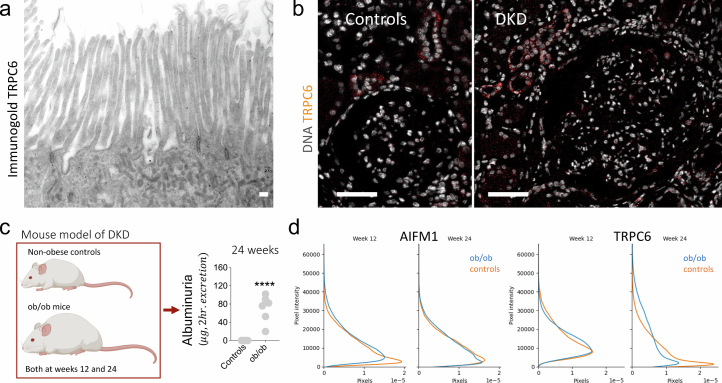
Validation of TRPC6 expression in experimental diabetic kidney disease. (a) Expression of TRPC6 in normal murine proximal tubuli using immunogold in electron microscopy. This is a representative image from 3 biological replicates. (b) Expression of TRCP6 using Imaging Mass Cytometry in the human kidney (controls and diabetic kidney disease; DKD). These are representative images from 2 biological replicates per condition. (c) Schematic representation of a murine model of obesity and early DKD, showing albuminuria at 24 weeks of age. (d) Increased expression (distributional shift in pixel intensity) of TRPC6 in proximal tubuli in ob/ob mice at 24 weeks of age. Scale bar in (a) represents 250 nm and scale bars in (b) represent 50 µm. Parts of panel c were created using BioRender (https://biorender.com).

Next, we sought to apply PathoPlex to unravel the complexities of human disease. The performance of PathoPlex was tested in clinical specimens with patient-level heterogeneity in one of the most common clinical features of end-organ damage in diabetes, namely DKD^38^. A total of 38 human kidney specimens (from 18 individuals without diabetes (controls) and 20 individuals with advanced DKD) were profiled in 422 ROIs using 61 markers (Supplementary Table 3) to obtain >100 billion pixels at 160 nm per pixel (Fig. 3a). PathoPlex identified 18 clusters with differential abundance between control and DKD samples (Supplementary Table 4). For example, cluster 19 (with contributors from apoptosis inducing factor mitochondria associated 1 (AIFM1) and transient receptor potential cation channel subfamily C member 6 (TRPC6)) was increased in tissues from individuals with DKD and localized primarily in the proximal tubules. This result was corroborated when projected onto conventional histopathology images (Fig. 3b). We validated the expression of TRPC6 in proximal tubules using both immunogold in electron microscopy (Extended Data Fig. 6a) and IMC-based antibody expression (Extended Data Fig. 6b). Analyses of a mouse model of DKD (Extended Data Fig. 6c) also confirmed that TRPC6 expression is increased in proximal tubules (Extended Data Fig. 6d).

**Extended Data Fig. 7:**
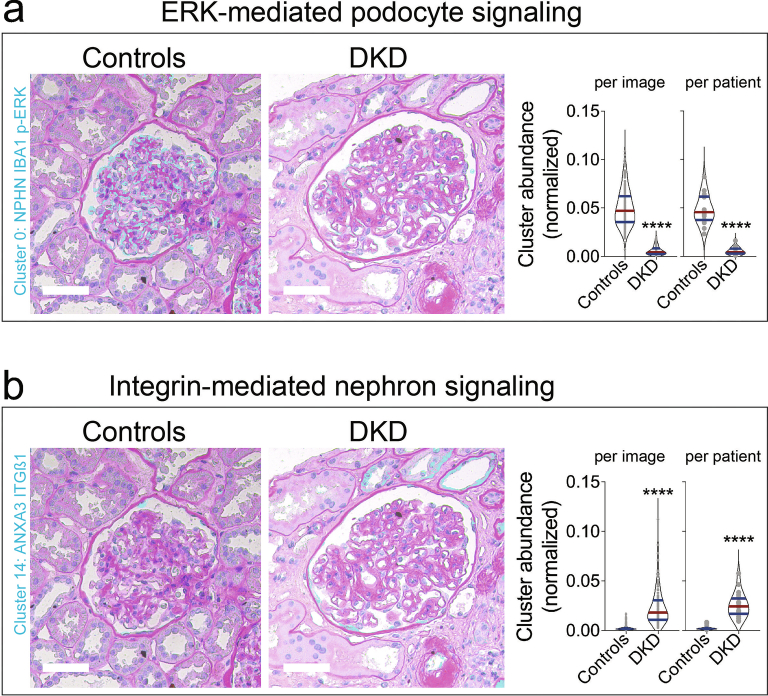
Projecting clusters onto histopathology staining. Histopathological staining allows cluster assignment to exact structural locations. (a) Cluster 0 (ERK-mediated podocyte signaling) and (b) cluster 14 (integrin-mediated nephron signaling). In both cases, cluster abundance was regulated in DKD. ****P < 0.0001. Scale bars represent 100 µm.

**Extended Data Fig. 8:**
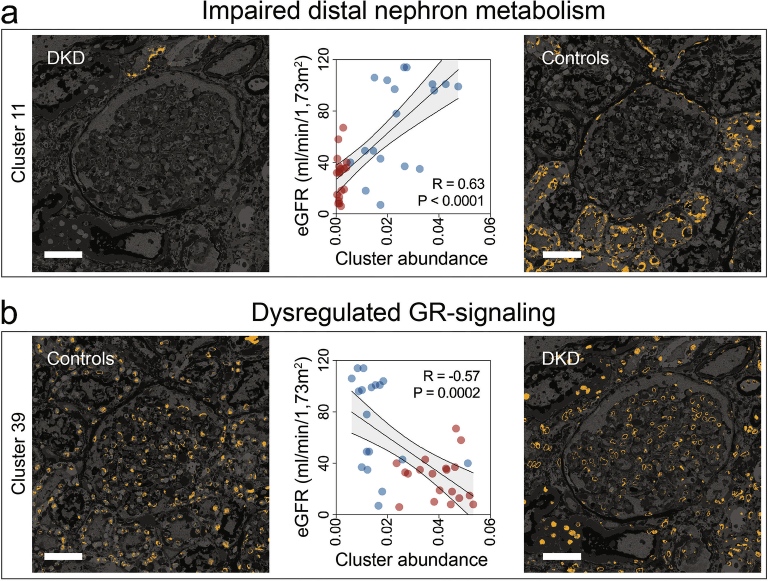
Cluster abundance associates with clinical kidney function. Cluster abundances correlate with clinical renal function (estimated glomerular filtration rate; eGFR). (a) Cluster 11 (impaired distal nephron metabolism) and (b) cluster 39 (dysregulated glucocorticoid receptor signaling). Scale bars represent 100 µm.

Our analysis revealed multiple additional differentially regulated clusters that reflected known biological processes, including ERK-mediated and integrin-mediated signalling in multiple nephron segments (Extended Data Fig. 7). To connect these findings to cell-level features, we performed deep-learning-based cellular segmentation using CellPose^39^ (Supplementary Fig. 10) to characterize the co-occurrence of multiple clusters in well-defined cell-level metaclusters. For example, metacluster 16, which was increased in DKD, contained clusters that represented multiple processes associated with proximal tubule injury (Fig. 3c). Notably, a subset of clusters showed strong correlations with kidney function (Extended Data Fig. 8), including cluster 28 (extracellular matrix (ECM) remodelling) (Fig. 3d), thereby linking subcellular pathological features to patient-level organ function.

## Computational cross-validation

**Extended Data Fig. 9:**
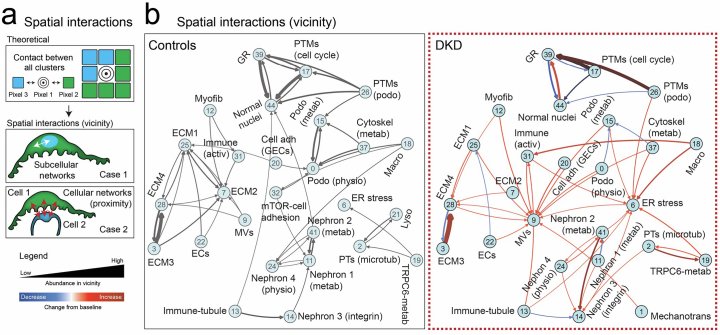
Multivariate cluster join counts analysis. (a) Schematic representation of our approach to characterize direct spatial interactions between neighbouring clusters. (b) Spatial interactions highlight changes in associations across disease states and scales. GR, Glucocorticoid receptor; PTMs, Post-translational modifications; PTs, proximal tubular cells; DT, distal tubule; CD, collecting duct; ER, Endoplasmic reticulum; MVs, microvesicles; ECM, Extra cellular matrix; activ, activation; podo, podocytes; physio, physiology; metab, metabolism; cytoskel, cytoskeletal; ECs, endothelial cells; GECs, glomerular endothelial cells; mTOR, mammalian target of Rapamycin; macro, macrophages; lyso, lysosomes; microtub, microtubules; mechanotrans, mechanotransduction; Cell adh, cell adhesion; Myofib, myofibroblasts.

**Extended Data Fig. 10:**
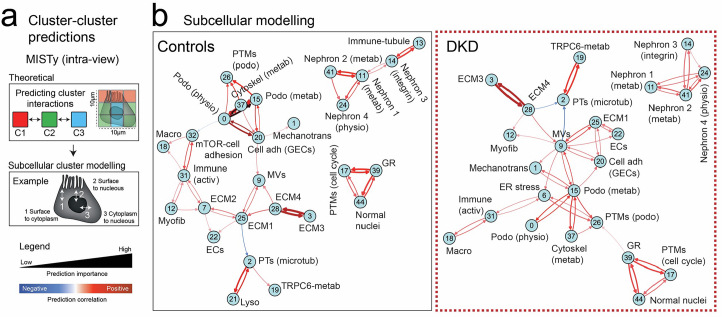
Condition-specific structural patterns. (a) Schematic representation of our approach to characterize cluster-cluster predictions using MISTy. (b) Cluster predictions highlight changes across disease states and scales. GR, Glucocorticoid receptor; PTMs, Post-translational modifications; PTs, proximal tubular cells; DT, distal tubule; CD, collecting duct; ER, Endoplasmic reticulum; MVs, microvesicles; ECM, Extra cellular matrix; activ, activation; podo, podocytes; physio, physiology; metab, metabolism; cytoskel, cytoskeletal; ECs, endothelial cells; GECs, glomerular endothelial cells; mTOR, mammalian target of Rapamycin; macro, macrophages; lyso, lysosomes; microtub, microtubules; mechanotrans, mechanotransduction; Cell adh, cell adhesion; Myofib, myofibroblasts.

**Extended Data Fig. 11:**
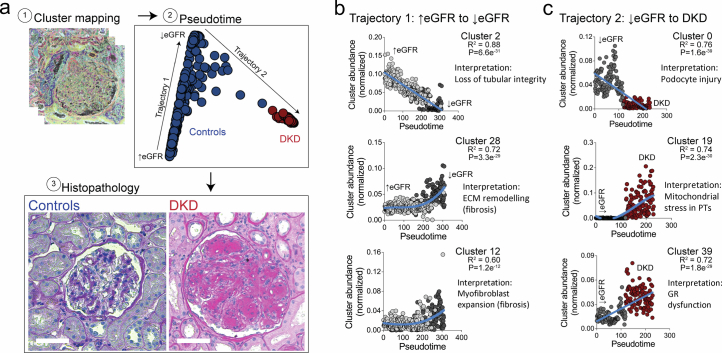
Image-level pseudotime analysis from cluster mapping using PILOT. (a) Pseudotime analysis was performed based on our interpretable clusters, identifying a path from controls to diabetic kidney disease (DKD) that correlates with histopathological changes. This path can be separated into two trajectories. (b) Trajectory 1 defined a transition based on estimated glomerular filtration rate (eGFR), marking the range in renal function in our non-diabetic controls. Pseudotime was strongly associated with clusters representing loss of tubular integrity, extracellular matrix remodelling (ECM; fibrosis) and myofibroblast expansion (fibrosis). (c) Trajectory 2 was strongly associated with clusters representing podocyte injury, mitochondrial stress in proximal tubuli (PTs) and glucocorticoid receptor (GR) dysfunction. PILOT uses non-linear regression methods and leverages the Wald test to evaluate the difference in the fitted model for each cluster vs. the model fitted for background clusters.

To further validate the biological relevance of PathoPlex-derived clusters, we calculated the multivariate cluster join counts for each biopsy sample independently and then averaged them at the condition level (Extended Data Fig. 9). These subcellular and intercellular spatial networks recapitulated aspects of kidney architecture by arranging them into functional compartments, including glomerular and tubular segments, individual cell types (for example, podocytes) and ECM. Moreover, the networks highlighted pathophysiological changes (for example, an increased connection between proximal tubule microtubules and Ca^2+^ signalling). Next, we applied a nonlinear co-occurrence prediction model across cell-sized windows (MISTy)^40^, which identified groups of condition-specific mutually predictive clusters that reflected functional (for example, glomerular, tubular and interstitial) and subcellular (for example, nuclear or cytoplasmic) compartments that defined immune activation, ECM remodelling, metabolic stress and cell injury (Extended Data Fig. 10). We also performed image-level pseudotime analysis with a multiscale model^41^ to propose a path from individuals without diabetes but with varying kidney function to individuals with DKD. This analysis resulted in the identification of two potential trajectories of pathogenesis that showed a strong association with histopathological changes (Extended Data Fig. 11a). For trajectory 1, determinant features included tubulointerstitial fibrosis, which reflected the loss of kidney function in a subpopulation of individuals without diabetes (Extended Data Fig. 11b). For trajectory 2, specific features included podocyte injury, Ca^2+^-mediated mitochondrial stress in proximal tubules and glucocorticoid receptor (GR) dysfunction, which reflected diabetic end-organ damage in individuals with impaired kidney function (Extended Data Fig. 11c).

To reinforce the value of PathoPlex as a foundational tool to perform unsupervised disease phenotyping, we used UnPaSt^42^ to conduct label-free biclustering based on cluster abundances. UnPaSt was able to accurately discriminate between control and DKD samples (Fig. 3e). Bicluster-specific clusters reflected the increased abundance of functional integrity features (that is, podocyte physiology and metabolism) in control specimens, and of pathogenic features in DKD samples (that is, macrophage infiltration, immune activation, AIFM1–TRPC6 signalling, endoplasmic reticulum (ER) stress, ECM remodelling, and GR, β-catenin, histone H2B and ubiquitylation dysfunction). In summary, PathoPlex-derived clusters can be immediately used to extend the scope of computational analyses to add layers of biological context (that is, pseudotime, niche profiling and feature subclassification) and to connect PathoPlex to the broader computational spatial biology ecosystem.

## Druggability profiling

**Extended Data Fig. 12:**
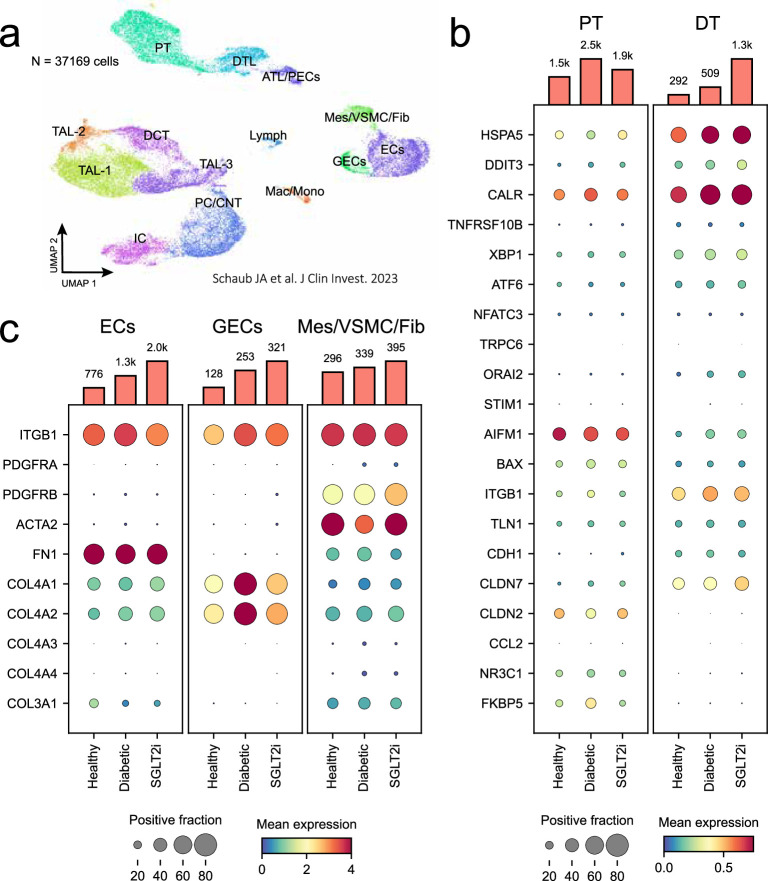
External validation of DKD features. (a) Cell types identified via single-cell RNA-sequencing. Even in early diabetic kidney disease, transcripts related to ER stress, Ca2+-mediated tubular injury, integrin beta 1-, and GR-signaling (b), as well as ECM remodelling and pro-fibrotic signaling (c) showed differential gene expression patterns compatible with differences observed at the protein level found in advanced diabetic kidney disease. SGLT2 inhibitor (SGLT2i)-treated patients exhibited incomplete modulation of transcriptomic changes. TAL: Thick ascending limb; PT: Proximal tubule; PC: Principal cell; ECs: Endothelial cells; IC: Intercalated cell; PC/CNT: Principal cells / Connecting tubule; DCT: Distal convoluted tubule; Mes/VSMC/Fib: Mesangial cells/Vascular smooth muscle cells/Fibroblasts; ATL/PEC: Ascending thin limb/Parietal epithelial cells; GECs: Glomerular endothelial cells; Mac/Mono: Macrophages/Monocytes; Lymph: Lymphocytes.

Next, we aimed to leverage PathoPlex-derived clusters to infer additional clinically relevant information, such as potential opportunities for drug repurposing. First, we selected the top contributing proteins from each cluster to define a cluster-based DKD signature that was extended using the search tool for the retrieval of interacting genes and proteins (STRING)^43^. Then we cross-referenced our extended DKD signature with the Comparative Toxicogenomics Database (CTD)^44^. Notably, different drug classes used in the standard treatment of diabetes^45^ interacted with the proteins represented in our extended DKD signature (Fig. 3f), including SGLT2 inhibitors^38^. Next, we analysed a publicly available single-cell RNA-sequencing dataset^46^ generated from recently diagnosed young individuals with T2D without overt DKD and included a subset of patients receiving an SGLT2 inhibitor. This analysis confirmed our extended DKD signature at the transcriptional level and revealed a partial transcriptional modulation in proximal tubules with SGLT2 inhibitor treatment (Extended Data Fig. 12 and Supplementary Table 5). This result suggests that individuals with diabetes may benefit from additional interventions to reverse them to the healthy reference state. For this reason, we quantified the number of known drug–protein interactions for members of our extended DKD signature. This analysis led to the identification of potential targets to revert cell communities to the healthy reference state, including phosphodiesterase-5 inhibitors as potential regulators of TRPC6 signalling. As an additional external validation step, we used a public single-nucleus RNA-sequencing dataset from a rat model of DKD^47^ to assess the link between cGMP signalling and TRPC6-mediated mitochondrial stress in proximal tubules (Fig. 3g). Although transcriptomic detection of TRPC6 was insufficient to confirm a direct effect on this target, cGMP modulation was associated with the attenuation of several components of our extended DKD signature. Together, our findings confirm that the applicability of PathoPlex-derived clusters extends beyond the definition of integrative pathological features. Indeed, they can link the spatial context to single-cell transcriptomics and even pharmacological modelling.

## Beyond classical pathology

**Fig. 4:**
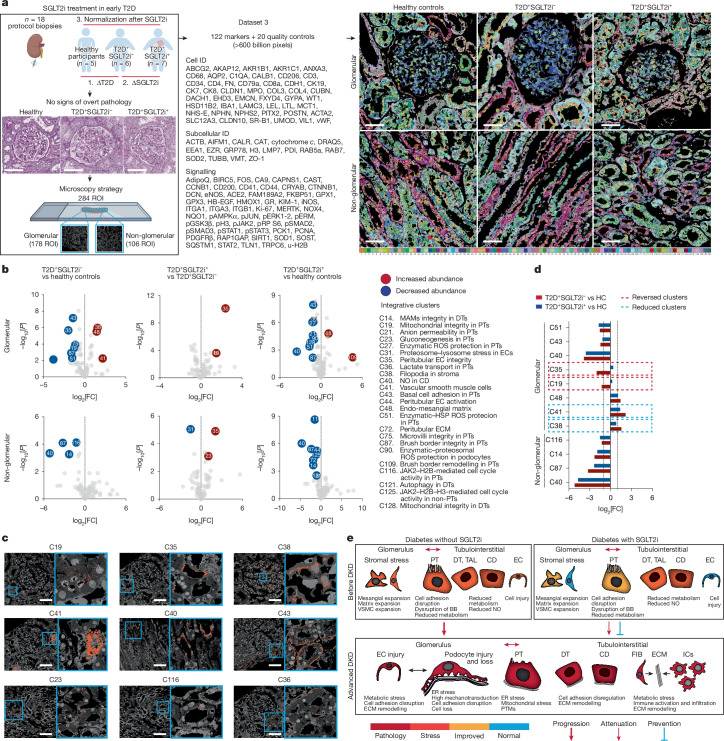
PathoPlex as a tool to decode kidney injury before DKD. **a**, Schematic overview showing the experimental design (*n* = 18 cases; ROIs = 284). Details of the antibody panel are provided in Supplementary Table 6. For this experiment, we used human research specimens from healthy individuals and individuals with T2D treated or not with SGLT2 inhibitors (SGLT2i). The images on the right show 140 clusters projected. **b**, Differential cluster abundance for each comparison. The key for clusters also applies to **c** and **d**. **c**, Examples of integrative subcellular clusters that were differentially regulated. Images were selected from all available ROIs (*n* = 284). **d**, Mean effect of SGLT2i on regulated clusters, showing examples of persistently dysregulated (C14, C40, C43, C48, C51, C87 and C116), statistically improved (C38 and C41) and normalized (C19 and C35) clusters. HC, healthy controls. **e**, Cluster-based model of glomerular and tubulointerstitial alterations before the development and in late stages of DKD, accounting for effects of SGLT2i. Differential cluster abundance analysis used a two-sided *t*-test with Benjamini–Hochberg correction. Scale bars, 100 µm (**a**,**c**). BB, brush border; CD, collecting duct; DT, distal tubule; EC, endothelial cell; FIB, fibroblasts; HSP, heat shock protein; IC, intercalated cell; MAM, mitochondria-associated endoplasmic reticulum membrane; NO, nitric oxide; ROS, reactive oxygen species; TAL, thick ascending limb; VSMC, vascular smooth muscle cell. Diagrams in **a** were created using BioRender (https://biorender.com). Source Data

Up to this point, our experiments included well-defined disease and control groups with recognizable pathological features identifiable through traditional histopathological methods. In our final experiment, PathoPlex was applied to 18 human kidney research biopsy samples without overt histopathological changes to test the limits and added value of PathoPlex. We aimed to identify early stages of kidney stress in T2D and to further profile the impact of SGLT2 inhibitors on these integrative features of cellular stress. To this end, archival tissue specimens from 5 healthy individuals, 6 individuals with T2D not treated with SGLT2 inhibitors (T2D^+^SGLT2i^–^) and 7 individuals with T2D treated with SGLT2 inhibitors (T2D^+^SGLT2i^+^) from a previous study^46^ were selected for analysis. A total of 142 markers (122 biological and 20 quality control; Supplementary Table 6) were imaged at 80 nm per pixel, which systematically covered glomerular and non-glomerular regions across 284 ROIs. This strategy generated >600 billion pixels, which contributed to 140 clusters (Fig. 4a). A total of 24 clusters showed significant regulation between groups (Fig. 4b), which revealed specific biological processes with distinct subcellular locations (Fig. 4c). Significant differences encompassed increases in clusters that represented stromal cell filopodia, the mesangial matrix and vascular smooth muscle cells. Moreover, reductions in clusters associated with structural and functional features of proximal tubules (that is, cell adhesion, brush border integrity, JAK2–H2B-mediated cell cycle, mitochondrial integrity and lactate transport), peritubular capillary integrity, mitochondrial and ER integrity in the distal tubule and nitric oxide production in the collecting duct were observed.

SGLT2 inhibitors attenuated changes in peritubular capillaries and mitochondrial integrity in proximal tubules, and increased gluconeogenesis in proximal tubules. SGLT2 inhibitors were also characterized by the decreased abundance in a cluster representing lysosomal and proteasomal stress in endothelial cells. However, SGLT2i treatment did not fully reverse the T2D-specific changes in cluster abundance. First, the fold changes of significantly differentially abundant clusters in T2D^+^SGLT2i^–^ samples relative to control samples were defined to represent the baseline effect of T2D. Then, the same comparison was performed between T2D^+^SGLT2i^+^ and control samples to represent the effect of SGLT2 inhibitors on T2D. This comparison indicated that SGLT2 inhibitors promoted a reconstitution of peritubular capillary and mitochondrial integrity in proximal tubules, together with a partial reversal of the increase in clusters representing vascular smooth muscle and stromal filopodia (Fig. 4d). These data demonstrate the potential of PathoPlex to uncover features of injury before disease onset that are inaccessible to classical histopathology.

Finally, on the basis of the two diabetes datasets generated in this study (Figs. 3 and 4), we propose a continuum of early glomerular and tubulointerstitial alterations that precede quantifiable reductions in end-organ function and that eventually converge in DKD. These alterations include an impaired glomerular filtration barrier, podocyte loss, ECM remodelling and tubular injury following prolonged hyperglycaemia (Fig. 4e). Although some early changes seemed to be attenuated by SGLT2 inhibitors, further studies are required to fully profile the potential long-term preventive effects of this intervention throughout the entire clinical course of DKD. In summary, our results demonstrate the utility of PathoPlex to extract meaningful integrative features from even non-pathological tissues. Notably, the framework also provided further pathophysiological evidence to support the use of SGLT2 inhibtors^31^ as an early intervention in T2D. Our results highlight the potential need for further treatments to optimally preserve kidney health in individuals with T2D at high risk of DKD.

## Discussion

Multiplexed imaging is a rapidly growing field^10,48^ and its contribution to a deeper understanding of tissue biology is illustrated by the recent generation of organ-level atlases, for example, in placenta^8^ and intestine^9^ using MIBI-TOF and CODEX, respectively. Moreover, previous efforts to characterize archival pathological tissues have provided new insights into the tumour microenvironment using IMC in breast cancer^49,50^ and melanoma^51^ as well as in post-mortem COVID-19 specimens using IBEX^52^. Despite these recent successes, widespread application of these technologies has been hampered by several factors. These include access to commercial equipment, antibody panel size (average of 37 markers) and composition (that is, mostly focused on cell identity), limited spatial resolution (average of 267 nm per pixel), high-throughput in single specimens and insufficiently defined quality control steps. Here we provided a detailed protocol for highly multiplexed imaging at subcellular resolution for archival FFPE tissues. The use of PathoPlex for multiplexed imaging includes the following advantages: (1) no dependency on commercial equipment; (2) open-access 3D printing-based solutions for sample preparation and automation; (3) compatibility with any inverted fluorescence microscope, ranging from widefield to high-end confocal microscopy; (4) scalability in antibody panel size (>120), image resolution (up to 80 nm per pixel using confocal microscopy) and sample sizes (that is, intact clinical tissues); (5) use of unmodified antibodies broadly accessible to the scientific community; (6) introduction of stringent quality-control steps to define best practices; and (7) minimization of batch effects through the parallel processing of up to 40 clinical biopsy samples (approximately 4,000 mm^2^ of available tissue). Together, PathoPlex paves the way for universal access to multiplexed imaging in clinical specimens. It also unlocks one of the largest and most comprehensive biobanks inadvertently created to a wide array of users: FFPE archives in clinical pathology centres and research institutes. It is now up to users to build on this resource to explore well-characterized patient cohorts, generate antibody panels that best address their scientific or clinical questions and to leverage the most efficient and suitable microscopy systems.

As emerging multiplexed imaging technologies generate larger and more complex datasets, image analysis tools need to adapt. Although cell segmentation and identification of cell states remain the most accepted methodologies for both technical development^53^ and biological interpretability^54^, unsupervised methods are starting to gain attention. Recent examples of pixel-based image analysis tools for multiplexed imaging data include cellular changes during normal retinal development in human organoids^16^ and the generation of quantitative annotations both independently and in conjunction with cell segmentations in various human tissues^55^. However, PathoPlex provides integrative features that recapitulate health, stress and overt disease, which can be pharmacologically modulated. As part of PathoPlex, we provide spatiomic, an efficient, scalable and streamlined end-to-end workflow for the community to analyse multiplexed imaging datasets of over half a trillion pixels. Overall, PathoPlex introduces a shift away from characterizing tissues solely at the cellular level (cell typing and their spatial organization) and towards a data-driven approach that captures the most distinctive biological signatures across spatial scales based on spatial co-expression patterns derived from individual pixels.

Recent advances in community-based strategies include minimum information guidelines^56^ and a public repository for antibodies compatible with multiplexed imaging^57^. These initiatives highlight the importance of continuous development in this new and rapidly growing field. Although PathoPlex shows promise, several areas require further optimization. PathoPlex enables users to perform more imaging cycles, which provides an opportunity to expand antibody panel sizes and in turn can extend their scope. However, time efficiency remains crucial for implementation in research and even more so as a clinical application. For this reason, we consider that robotic automation or sample size enrichment through parallel processing of multiple tissue microarrays may be considered in the future. Furthermore, as antibody panels can rapidly expand, standardizing quality-control metrics (that is, validation, secondary-only cycles and re-staining) will benefit potential PathoPlex users and the growing multiplexed imaging community. Moreover, PathoPlex enables the generation of datasets with sizes beyond current standards (>600 billion pixels), which present an analytical challenge that is currently best addressed by GPU acceleration. In this context, we recognize the need to implement additional features to minimize user reliance on specialized hardware. Finally, our work raises important computational questions regarding the need to establish integrative ontology terms that combine multiple biological layers to facilitate broad interpretability. Moreover, the increasing technical requirements to transfer, share, store and process data at a scale will soon challenge available resources.

## Methods

### Archival tissues

#### Human samples

FFPE tissues were collected and prepared according to institutional protocols. For Fig. 2, validation kidney biopsy specimens from individuals with ANCA-associated CGN were obtained from the Hamburg Glomerulonephritis Registry (https://www.sfb1192.de/en/register). For Fig. 3, control kidney specimens were obtained from nephrectomies performed on individuals with renal cell carcinoma in collaboration with the Division of Nephrology and Clinical Immunology, RWTH Aachen University Medical Center. Kidney biopsy samples from individuals with DKD were obtained from the Department of Nephrology and the Department of Pathology Georges Pompidou European Hospital, Assistance Publique–Hôpitaux de Paris. For Fig. 4, nested protocol research kidney biopsy samples were obtained from volunteers (adolescents and young adults; *n* = 13) with T2D (12–21 years of age, T2D onset at <18 years of age, T2D duration 1–10 years and HbA1c < 11%) from the Renal HEIR and the IMPROVE-T2D studies. The participants were recruited from the Type 2 Diabetes and Metabolic Bariatric Surgery clinics at the Children’s Hospital Colorado Anschutz Medical Campus in Aurora. T2D was defined according to criteria of the American Diabetes Association plus the absence of glutamic acid decarboxylase, islet cell, zinc transporter 8 and/or insulin autoantibodies. The Renal HEIR and IMPROVE-T2D cohorts have intentionally harmonized study protocols. Medication use was recorded for all participants, and T2D treatment, including SGLT2 inhibitors, was determined by their medical provider. Normative kidney reference tissue from research biopsy samples were provided by five healthy young adult participants in the CROCODILE study (NCT04074668). For Supplementary Figs. 3 and 4, kidney biopsy samples were obtained from the Hamburg Glomerulonephritis Registry (https://www.sfb1192.de/en/register), liver specimens were provided by the Institute of Pathology, University Medical Center Hamburg-Eppendorf and brain specimens were provided by the Institute of Neuropathology, Freiburg University Hospital. Ethics approvals were obtained from the Institutional Review Board of the RWTH Aachen University Medical Center (EK-016/17), the local ethics committees of the Chamber of Physicians in Hamburg (PV4806) and Freiburg (Ethikvotum 10008/09), the Paris Ethics Committee (IRB00003888, FWA00005831) and the Colorado Ethics Committee (NCT03584217 and NCT03620773). All tissue collections were performed in accordance with the ethical principles stated by the Declaration of Helsinki.

#### Rodent samples

Archival FFPE tissues from experimental immune-mediated kidney disease and DKD were collected according to institutional protocols of Hamburg, Melbourne, Heidelberg and Paris (N047/20, MMCB/2006/29, H2052-2071/23 and 358-86/609EEC, respectively). All experimental animals were housed at an ambient temperature of 20 ± 2 °C, humidity of 55 ± 10% and a light–dark cycle of 12–12 h. In brief, mouse crescentic nephritis was induced according to an established protocol^58^. Rat tissues were obtained from two experimental set-ups^32,59^. Administration of a JNK inhibitor (CC930, dose of 60 mg kg^–1^ in 0.5% carboxymethyl cellulose) or vehicle alone was performed twice daily by oral gavage. The prevention study (therapy started at day 0 and animals were killed on day 1) was performed in outbred male Sprague–Dawley rats, as this strain is known to develop heavy proteinuria^59^. The therapeutic study (therapy started at day 7 after disease induction and continued until animals were killed on day 28) was performed in inbred male Wistar Kyoto rats, which are prone to developing crescent formation. In both studies, proteinuria measurements and histopathology were performed according to standardized protocols^32,59^. *Btbr-Lep*^*ob/ob*^ (*Btbr*^*ob/ob*^) mice were obtained by crossing two heterozygous *Btbr*^*ob/WT*^ mice purchased from The Jackson Laboratory. This model shows morphological and physiological traits of DKD (that is, hyperglycaemia, albuminuria and glomerular hypertrophy). Wild-type littermates were used as controls.

### Highly multiplexed imaging

#### Sample preparation

Depending on the number of samples, a suitable-sized glass surface was selected and coated with poly-d-lysine (1 mg ml^–1^; Merck, A-003-E) for 30 min or with APTES (Merck, 440140) 10% v/v in acetone (Merck, 320110) for 2 min and then dried overnight before mounting the sections. We initially used poly-d-lysine for all our experiments but realized that significant lifting was progressively observed in all organs tested, including kidney, lung, colon, liver and brain. Lifting was initially mild in kidney samples but highly prominent in lung and colon specimens. For example, we observed partial but meaningful tissue lifting in 76 out of 498 ROIs (15%) by the end of 49 imaging cycles (Fig. 3). Furthermore, from 23 lung specimens analysed over 8 imaging cycles, lifting was already observed in 7 of them (30%). These observations across multiple tissue types led us to conclude that poly-d-lysine coating exhibits organ-dependent and time-dependent reliability limitations, for which we recommend potential users to perform pilot studies in their organ of interest. However, after a comprehensive literature review, we identified APTES as an ideal coating agent. Using APTES, tissue lifting occurred in only 1% of kidney specimens (Fig. 4). After coating, FFPE tissues were cut at a thickness of 2–3 μm and carefully mounted on the coated glass surface (for example, µ-Slide 2-well glass-bottom (Ibidi, 80287), µ-Slide 8-well glass-bottom (Ibidi, 80827), Cell Imaging Plate 24-well glass-bottom (Eppendorf, 0030741021) or Nexterion glass (Schott, 1868767)). To prevent dissolution of the plastic components in the chambered coverslips and plates by the solvent used for deparaffinization, the walls of each well were protected by a seal of transparent silicone (Pattex) or a ring of solvent-resistant plastic, respectively.

The following steps were performed only once before initiating the sequence of cycles.

#### Deparaffinization and rehydration

Samples were treated with the following set of solutions: Histo-Clear (National Diagnostic, HS-200) three cycles of 10 min each, followed by an ethanol series consisting of three cycles of 100% ethanol (10 min), two cycles of 70% ethanol (5 min), one cycle of 50% ethanol (5 min) and finally, triple immersion in double-deionized water (ddH_2_O) for 5 min each.

#### Antigen retrieval

Samples were immersed in target retrieval solution pH 9 (Agilent, S236784-2) and heated for 15 min using a steamer (Braun; FS 3000). Afterwards they were left to cool down to room temperature for 30–60 min. Sections were then incubated for 15 min in EnVision FLEX wash buffer (Agilent, K800721-2).

#### Blocking

To limit nonspecific antibody binding, samples were incubated in a blocking solution consisting of 5% BSA (Merck, A7906) in Dulbecco’s PBS (Thermo Fisher Scientific, 14190094) for 1 h at room temperature. Afterwards, samples were washed three times for 5 min with wash buffer.

#### Elution

An elution buffer was prepared according to a previously described formulation^15^, which consisted of 0.5 M glycine (Carl Roth, 3908.2), 3 M urea (Merck, U5378), 3 M guanidine hydrochloride (Merck, G4505) and 70 mM TCEP (Merck, C4706) mixed in ddH_2_O and adjusted to pH 2.5. Samples were incubated in elution buffer once for 5 min and then three times for 10 min on a shaker, followed by three washes of 5 min with wash buffer.

#### NHS-E labelling

Whenever used as a reference for alignment, NHS-E (Thermo Fisher Scientific, A10168) diluted in PBS (1:400) was added to the samples for 1 h at room temperature. After 1 h, samples were washed three times for 5 min with wash buffer.

The following steps were completed for every subsequent cycle of staining and carried out in a light-free environment to prevent the crosslinking of antibodies.

#### Primary antibody stain for indirect immunofluorescence

Samples were incubated with primary antibodies in EnVision FLEX antibody diluent (Agilent, K800621-2) for either 1 h at room temperature (Fig. 2) or overnight at 4 °C (Figs. 3 and 4), followed by three washes of 5 min with wash buffer. We provide confirmation of each staining pattern for every antibody in Supplementary Data 1 and 2. We also validated the practical feasibility of 1-h incubations at room temperature under non-multiplexed and multiplexed imaging conditions (Supplementary Fig. 11).

#### Secondary antibody and nuclear stain for indirect immunofluorescence

Appropriately matched secondary antibodies (and directly conjugated primary antibodies) and the nuclear markers DAPI (Merck, D9542, 1:200) or DRAQ5 (Abcam, ab108410, 1:200) were mixed in antibody diluent and incubated for 1 h at room temperature. Afterwards, samples were washed three times for 5 min with wash buffer.

#### Imaging

An imaging buffer was prepared according to a previously described formulation^15^, which consisted of 700 mM *N*-acetyl-l-cysteine (Merck; A9165) mixed in ddH_2_O and adjusted to pH 7.4. Imaging buffer was added to samples for imaging and then washed three times for 5 min with wash buffer before elution.

#### Antibody elution

Samples were incubated with elution buffer once for 5 min and then three times for 10 min on a shaker, followed by three washes of 5 min with wash buffer.

Thereafter, these steps were repeated until the desired number of antibodies was reached. Together, each cycle (using 1 h of incubation time for primary antibodies) can be completed in under 4 h of bench work. All cycles per experiment (antibodies and order) are described in Supplementary Table 7. Periodic acid Schiff (PAS) staining was performed after the last immunofluorescence staining only in Fig. 3 following a standard protocol, including incubation with periodic acid (Th. Geyer, 3257.1) to oxidize the sections, followed by Schiff’s reagent (Merck, 1090330500) to label glycol-containing structures. The sections were then counterstained with Mayer’s haematoxylin (Agilent Technologies, S330930-2).

### Primary antibodies and lectins

#### For human samples

ABCG2 (Santa Cruz, sc-377176; 1:200); ACE-2 (R&D Systems, AF933; 1:200); adiponectin (Thermo Fisher Scientific, MA1-054; 1:200); AIF (Cell Signaling Technology, 5318; 1:200); AKAP12 (Proteintech, 25199-1-AP; 1:600); AKR1B1 (Thermo Fisher Scientific, PA5-82915; 1:500); AKR1C1 (Thermo Fisher Scientific, MA5-32842; 1:200); alpha B-crystallin (Proteintech, 68001-1-Ig; 1:1,000); ANXA3 (Sigma-Aldrich, HPA013398; 1:200); αSMA–FITC conjugate (Sigma-Aldrich, F3777; 1:800); aquaporin 2 (Alomone Labs, AQP-002; 1:400); β-actin (Sigma-Aldrich, A5441; 1:1,500); β-catenin (Abcam, ab6302; 1:2,000); β-tubulin (Cell Signaling Technology, 2128; 1:150); calbindin-D (Sigma-Aldrich, C9848; 1:3,000); calpain small subunit 1 (Abcam, ab92333; 1:200); calpastatin (Abcam, ab244460; 1:200); calreticulin (Abcam, ab92516; 1:300); carbonic anhydrase IX (R&D Systems, AF2188; 1:50); catalase (Proteintech, 66765-1-Ig; 1:300); CD3 (Abcam, ab11089; 1:200); CD4 (R&D Systems, AF-379-NA; 1:100); CD8 (Agilent, M710301-2; 1:200); CD34 (Agilent, GA63261-2; 1:50); CD41 (Thermo Fisher Scientific, PA5-79526; 1:500); CD42b (Abcam, ab227669; 1:100); CD44 (Cell Signaling Technology, 5640S; 1:200); CD44–Alexa Fluor 647 conjugate (BioLegend, 103018; 1:200); CD68 (BioLegend, 916104; 1:200); CD79α (Agilent, M705001-2; 1:200); CD200 (R&D Systems, AF2724; 1:100); CD206 (Proteintech, 60143-1-Ig; 1:2,000); FOS (Abcam, ab190289; 1:600); claudin 1 (Abcam, ab15098; 1:500); claudin 10 (Thermo Fisher Scientific, 38-8400; 1:100); collagen I (Southern Biotech, 1310-01; 1:200); collagen III (Abcam, ab7778; 1:200); collagen IV (Abcam, ab6586; 1:200); collagen V (Abcam, ab7046; 1:100); cubilin (R&D Systems, AF3700; 1:200), cyclin B1 (Cell Signaling Technology, 12231; 1:100); cytochrome *c* (Abcam, ab110325; 1:200); cytokeratin 7 (Agilent, GA61961-2; 1:300); cytokeratin 8 (R&D Systems, MAB3165-SP; 1:300); cytokeratin 19 (Abcam, ab52625; 1:300); C1QA (Proteintech, 67063-1-Ig; 1:1,000); DACH1 (Sigma-Aldrich, HPA012672; 1:200); decorin (R&D Systems, AF143, 1:50); E-cadherin (R&D Systems, AF648; 1:200); EEA1 (Santa Cruz, sc-137130; 1:100); EHD3 (LSBio, LS-C133741; 1:150); endomucin (Sigma-Aldrich, HPA005928; 1:100); eNOS (Abcam, ab76198; 1:200); ezrin (Cell Signaling Technology, 3145S; 1:300); FAM189A2 (Thermo Fisher Scientific, PA5-63414; 1:200); fibronectin (Abcam, ab2413; 1:200); FKBP51 (R&D Systems, AF4094-SP; 1:50); FXYD4 (Thermo Fisher Scientific, PA5-63570; 1:200); GFAP (Thermo Fisher Scientific, 14-9892-82; 1:200); glucocorticoid receptor (Cell Signaling Technology, 3660; 1:2,000); glutathione peroxidase 1 (R&D Systems, AF3798; 1:100); glutathione peroxidase 3 (R&D Systems, AF4199; 1:50); glycophorin A (R&D Systems, MAB1228-SP; 1:500); GRP78 (Proteintech, 11587-1-AP; 1:200); HB-EGF (R&D Systems, AF-259; 1:100); histone H3 (Cell Signaling Technology, 4499; 1:400); HMOX1 (Thermo Fisher Scientific, MA1-112; 1:200); HSD11B2 (R&D Systems, MAB8630-SP; 1:100); KIM-1 (R&D Systems, AF1750; 1:200); IBA1 (Thermo Fisher Scientific, MA5-27726; 1:500); IDH1 R132H (Dianova, DIA-H09, 1:200); IL-1RA (Abcam, ab124962; 1:200; specificity issues were raised by the provider after our experiments were completed. We kept it in the panel as none of our findings were affected and we did not perform any biological inferences on the basis of this antibody); iNOS (Thermo Fisher Scientific, MA5-41652; 1:200); integrin-α1 (R&D Systems, AF5676; 1:300); integrin-α3 (Proteintech, 66070-1-Ig; 1:2,000); integrin-β1 (Abcam, ab179471; 1:800); Ki-67 (Agilent, M724029-2; 1:200); laminin (Abcam; ab11575, 1:200); LAMP1 (Cell Signaling Technology, 9091; 1:300); LC3B (Cell Signaling Technology, 3868; 1:300); LEL-DyLight 649 conjugate (Vector Laboratories, DL-1178; 1:300); LTL biotinylated (Vector Laboratories, B-1325-2; 1:500); MCT1 (Thermo Fisher Scientific, MA5-18288; 1:300); MerTK (R&D Systems, AF591; 1:200); MPO (R&D Systems, MAB3174; 1:200); nephrin (Progen, GP-N2; 1:150); neurofilament (Agilent, IR607; 1:200); NOX4 (R&D Systems, MAB8158;, 1:300); NQO1 (Proteintech, 67240-1-Ig; 1:2500); OLIG2 (Bio SB, BSB 2561; 1:200); p62 (Cell Signaling Technology, 39749; 1:400); PCK1 (Proteintech, 66862-1-Ig; 1:400); PCNA (Abcam, ab29; 1:2,000); PDGFRβ (Cell Signaling Technology, 3169; 1:100); PDI (Cell Signaling Technology, 45596S; 1:400); periostin (R&D Systems, AF3548; 1:150); phospho-AMPKα (Cell Signaling Technology, 2535; 1:200); pJUN (Abcam, ab32385; 1:200); phospho-ERK1/2 (Cell Signaling Technology, 4370; 1:250); phospho-ezrin–radixin–moesin (Cell Signaling Technology, 3726; 1:200); phospho-GSK3β (Cell Signaling Technology, 9323; 1:100); phospho-histone H3 (Cell Signaling Technology, 9701; 1:200); phospho-JAK2 (Thermo Fisher Scientific, MA5-42424; 1:100); phospho-ribosomal protein S6 (Cell Signaling Technology, 4858S; 1:300); phospho-SMAD2 (Thermo Fisher Scientific, 44-244G; 1:200); phospho-SMAD3 (Thermo Fisher Scientific, PA5-104940; 1:200); phospho-STAT1 (Cell Signaling Technology, 9167S; 1:400); phospho-STAT3 (Abcam, ab76315; 1:200); PITX2 (R&D Systems, AF7388; 1:100); podocin (Sigma-Aldrich, P0372; 1:3,000); proteasome 20S LMP7 (Abcam, ab3329; 1:400); RAB5A (Cell Signaling Technology, 46449; 1:300); RAB7 (Abcam, ab137029; 1:200); RAP1GAP (Abcam, ab244259; 1:300); RCAS1 (Cell Signaling Technology, 12290; 1:200); sclerostin (Thermo Fisher Scientific, PA5-37943; 1:100); SIRT1 (Cell Signaling Technology, 8469; 1:200); SLC12A3 (Thermo Fisher Scientific, MA5-41643; 1:200); SOD1 (Proteintech, 67480-1-Ig; 1:400); SOD2 (Thermo Fisher Scientific, PA5-30604; 1:300); SRB1 (Abcam, ab217318; 1:300); STAT2 (R&D Systems, MAB16661; 1:200); survivin (Cell Signaling Technology, 2808; 1:300); talin 1 (Abcam, ab71333; 1:200); TRPC6 (Abcam, ab233413; 1:200); ubiquityl-histone H2B (Cell Signaling Technology, 5546T; 1:200); uromodulin (R&D Systems, AF5144; 1:300); villin 1 (Abcam, ab52102; 1:200); vimentin (Progen, GP53; 1:200); von Willebrand factor (Agilent, A008229-2; 1:200); WT1 (Agilent, IS05530-2; 1:200); and ZO-1 (Thermo Fisher Scientific, 61-7300; 1:250).

#### For mouse samples

ACE-2 (R&D Systems, AF933; 1:200); AIF (Cell Signaling Technology, 5318; 1:200); AKAP12 (Proteintech, 25199-1-AP; 1:600); ANXA3 (Sigma-Aldrich, HPA013398; 1:200); αSMA-FITC conjugate (Abcam, F3777; 1:800); aquaporin 2 (Alomone Labs, AQP-002; 1:400); calreticulin (Abcam, ab92516; 1:300); caspase 1 p20 (Thermo Fisher Scientific, PA5-99390; 1:200); CD3 (Abcam, ab1108; 1:200); CD4 (Abcam, ab183685; 1:200); CD41 (Thermo Fisher Scientific, PA5-79526; 1:500); CD42b (Abcam, ab227669; 1:100); CD44-Alexa Fluor 647 conjugate (BioLegend, 103018; 1:200); CD45 (Cell Signaling Technology, 70257; 1:200); FOS (Abcam, ab190289; 1:600); collagen I (Southern Biotech, 1310-01; 1:200); collagen IV (Abcam, ab6586; 1:200); cytochrome *c* (Abcam, ab110325; 1:200); DACH1 (Sigma-Aldrich, HPA012672; 1:200); E-cadherin (R&D Systems, AF648; 1:200); endomucin (Santa Cruz, sc-65495; 1:200); fibronectin (Abcam, ab2413; 1:200); histone H3 (Cell Signaling Technology, 4499; 1:400); IBA1 (Thermo Fisher Scientific, MA5-27726; 1:500); IL-1RA (Abcam, ab124962; 1:200; specificity issues were raised by the provider after our experiments were completed. We kept it in the panel as none of our findings were affected and we did not perform any biological inferences on the basis of this antibody); Ki-67 (Abcam, ab15580; 1:200); lamin B1 (Santa Cruz, sc-374015; 1:200); laminin (Abcam, ab11575; 1:200); LTL biotinylated (Vector Laboratories, B-1325-2; 1:500); nephrin (Progen, GP-N2; 1:150); PCNA (Abcam, ab29; 1:2,000); PDI (Cell Signaling Technology, 45596S; 1:400); phospho-ezrin–radixin–moesin (Cell Signaling Technology, 3726; 1:200); podocin (Sigma-Aldrich, P0372; 1:3,000); podoplanin (R&D Systems, AF3244-SP; 1:200); synaptopodin (Synaptic Systems, 163 004; 1:200); tyrosine hydroxylase (Cell Signaling Technology, 45648; 1:200); ubiquityl-histone H2B (Cell Signaling Technology; 5546T; 1:200); β-actin (Sigma-Aldrich; A5441, 1:1500); vimentin (Progen, GP53; 1:200); and von Willebrand factor (Agilent, A008229-2; 1:200).

### Secondary antibodies and biotin-binding proteins

Secondary antibodies were diluted in a ratio ranging from 1:200 to 1:300. The following antibodies were used: goat anti-guinea pig IgG Alexa Fluor 488 (Thermo Fisher Scientific, A-11073); goat anti-guinea pig IgG Alexa Fluor 555 (Thermo Fisher Scientific, A-21435); donkey anti-mouse IgG Alexa Fluor 488 (Thermo Fisher Scientific, A-21202); donkey anti-mouse IgG Alexa Fluor 555 (Thermo Fisher Scientific, A-31570); donkey anti-mouse IgG Alexa Fluor 647 (Thermo Fisher Scientific, A-31571); donkey anti-rabbit IgG Alexa Fluor 488 (Thermo Fisher Scientific, A-21206); donkey anti-rabbit IgG Alexa Fluor 555 (Thermo Fisher Scientific; A-31572); donkey anti-rabbit IgG Alexa Fluor 647 (Thermo Fisher Scientific, A-31573); donkey anti-goat IgG Alexa Fluor 488 (Thermo Fisher Scientific, A-11055); donkey anti-goat IgG Alexa Fluor 555 (Thermo Fisher Scientific, A-21432); donkey anti-rat IgG Alexa Fluor 488 (Thermo Fisher Scientific, A-21208); donkey anti-rat IgG Alexa Fluor 555 (Thermo Fisher Scientific, A78945); donkey anti-sheep IgG Alexa Fluor 488 (Thermo Fisher Scientific, A-11015); donkey anti-sheep IgG Alexa Fluor 555 (Thermo Fisher Scientific, A-21436); streptavidin Alexa Fluor 488 (Thermo Fisher Scientific, S11223); and streptavidin Alexa Fluor 555 (Thermo Fisher Scientific, S21381).

### Immunofluorescence in rat and human specimens

FFPE tissues were cut at a thickness of 2–3 μm, carefully affixed onto Superfrost Plus adhesion slides (Epredia, J1800AMNZ) and dried overnight at 37 °C. Subsequently, samples underwent sequential treatment involving triple immersion in xylene (10 min each) followed by an ethanol series (5 min each) consisting of three rounds of 100% ethanol, two rounds of 70% ethanol, one round of 50% ethanol and finally triple immersion in ddH_2_O (5 min each). The immunostaining procedure mirrored the one used for PathoPlex samples but substituted 5% BSA with SuperBlock blocking buffer (Thermo Fisher Scientific, 37535) during the blocking step. Finally, after immunostaining, samples were mounted using ProLong Gold (Thermo Fisher Scientific, P36930).

### Microscopy systems

For Fig. 2, images were acquired using a LSM 800 confocal microscope plus AiryScan (Zeiss, ZEN2.6) with the optimized ×63 objective (NA: 1.4). For Fig. 3, a Thunder Imager Live Cell and 3D assay (Leica Microsystems) fitted with a ×40 (NA: 1.10) or ×63 (NA: 1.40) objective was used to acquire images, which were processed using a computational clearing algorithm (Leica Microsystems)^60^. The positional data of the imaged region for each sample were stored in Leica Application Suite X software (v.3.7.6, Leica Microsystems), which ensured consistent capture of the identical location for each cycle. For Fig. 4, a CellDiscoverer 7 with LSM 900 (Zeiss, ZEN 3.5 System) and AiryScan Multiplex fitted with a ×50 (NA: 1.20) objective and ×0.5 zoom was used to acquire images. Supplementary Table 8 summarizes the approximate microscopy times per experiment, considering image acquisition as the most important contributing factor. However, there are additional practical contributors, including chamber repositioning, movement delay between the ROI and data storage, which should be accounted for during implementation.

### 3D printing

Tinkercad (Autodesk; https://www.tinkercad.com) was used to create designs for the 3D-printed parts. The design of the headpiece was adjusted on the basis of a previously proposed design^61^. The BLTouch Cover Size Fixed was designed by louise_tguk on Thingiverse (https://www.thingiverse.com/thing:5013058). The chamber frame, the table for the chamber frame, the corner frame, the stage, the solution container, stands 1 and 2 for the solution container, the discard container, the base plate, the headpiece, the alignment guide, the BLTouch cover size fixed and the BLTouch cover box were printed using PLA filament 1.75 mm (Flashforge). The inner frame was printed using NinjaFlex TPU filament 1.75 mm (NinjaTek). The dewaxing container, the dewaxing container holder, the dewaxing carrier and the carrier handle were printed using PolyLite PETG filament 1.75 mm (Polymaker). An Ender-5 Plus printer (Creality) was used. The following settings were implemented in Ultimaker Cura (v.4.13.1; Ultimaker): nozzle size, 0.40 mm; layer height, 0.20 mm; wall thickness, 2.0 mm (PLA and PETG for containers), 1.2 mm (PLA and PETG for others) and 0.80 mm (TPU); top and bottom thickness, 2.0 mm (PLA and PETG for containers), 0.8 mm (PLA and PETG for others and TPU); nozzle temperature, 190 °C (PLA) and 235 °C (PETG and TPU); bed temperature, 69 °C (PLA), 75 °C (PETG) and 50 °C (TPU); fan speed, 100%; print speed, 60 mm s^–1^ (PLA), 40 mm s^–1^ (PETG) and 20 mm s^–1^ (TPU); first-layer print speed, 20 mm s^–1^ (PLA), 15 mm s^–1^ (PETG) and 10 mm s^–1^ (TPU); infill, 20% and zigzag; build-plate adhesion, brim. Masking tape was used to create an adhesive surface on the bed.

### 3D printer-based liquid-handling system

To prepare for the use of the liquid-handling system, several preliminary steps were required. These encompassed manual deparaffinization, antigen retrieval and mounting the Nexterion glass with sample sections onto the chamber frame. The deparaffinization process described above required the use of a dewaxing container, a container holder, a dewaxing carrier and carrier handle printed with PETG. After completion of dewaxing, the Nexterion glass with sections underwent antigen retrieval and washing procedures as outlined above. After washing, any excess wash buffer present at the edges of the glass was carefully removed. The Nexterion glass was then inserted into the bottom of the chamber frame, and its edges were securely sealed using silicone. It was crucial to allow the silicone to dry for a minimum of 15 min. To prevent the samples from drying out during this process, regular application of wash buffer to the samples was necessary while ensuring that the silicone did not become excessively wet. Once the silicone was completely dry, the inner frame was positioned in the frame and samples were covered with wash buffer. The following process used a liquid-handling system based on a 3D printer (Ender-5 Plus). For light shielding, the 3D printer was covered by a 3D Printer enclosure (Creality). The window on the front of the enclosure needed to be covered with an opaque material to shield the inside from light. The BLTouch built into the Ender-5 Plus was also partially shielded by attaching the BLTouch Cover Size Fixed. Our liquid-handling system was based on three different g-codes: (1) ‘BSA to Elu.gcode’, which automated the process from blocking with BSA during the initial cycle to elution and the pouring of imaging buffer; (2) ‘1st Ab to Img.gcode’, which automated the process of washing the imaging buffer, incubating the primary and secondary antibodies and pouring the imaging buffer; and (3) ‘Elu to Img.gcode’, which automated the process of washing the imaging buffer, elution and pouring the imaging buffer. The settings of the solution containers corresponding to each g-code are shown in Supplementary Table 9. The solution container stand consists of numbered sections ranging from 1 to 12, which are designated for container installation. Each solution-filled container was placed in the section with the corresponding number on the stand. The corner frames were inserted into the holes at the four corners of the table. Solution container stands 1 and 2 with solution containers, the discard container, the stage, the table and the chamber frame were placed on the base plate. Specifically, solution container stand 1 needed to be positioned at the front side of the base plate. To prepare for the operation of the 3D printer, the print bed was removed and autolevelling was disabled. Once each g-code was initiated, after calibrating the home position, the printer head moved slightly backward and the printer stage was lowered. The printer then paused for 60 s before resuming operation. During this pause, the headpiece and BLTouch cover box were attached to the printer head and BLTouch, respectively, and the base plate, complete with all the necessary components and solutions, was placed on the printer stage using the alignment guide. Once installation was complete, the enclosure was completely closed. The washing, staining and elution processes were then automatically performed by pushing down on the solution containers and table with a rod in the headpiece. The BSA to Elu.gcode, 1st Ab to Img.gcode and Elu to Img.gcode programs were completed in approximately 2 h 15 min, 3 h 10 min and 1 h 15 min, respectively. The dimensions of the chamber frame match those of ready-made plates used for imaging cultured cells. For an example of how this solution works, see Supplementary Video 1.

### PEC cell line for migration assays

Primary PECs were thawed and cultured at 5% CO_2_ and 37 °C in endothelial cell basal medium (ECBM; PromoCell, C-22210) and 20% FBS (Thermo Fisher Scientific; 10500064) until 70%–80% confluence. The maintenance culture was passaged three times a week by gentle trypsinization using trypsin-EDTA 0.05% (Thermo Fisher Scientific, 25300054).

Migration assays were performed using Culture-Insert 2 wells in μ-Dish 35 mm (Ibidi, 81176). Each well was seeded with 30,000 PECs in 100 μl ECBM without supplements and with 1% FBS and incubated overnight. The insert was then removed, which created a gap of 500 μm between cells. PECs were stimulated with either PDGF-BB (Thermo Fisher Scientific, 315-18-50UG, per manufacturer’s recommendations of 2.0 ng ml^–1^) or with PDGF-BB and tanzisertib HCl (CC-930; Selleck Chemicals, S8490) or PDGF-BB and vehicle, in this case DMSO (Merck; D2650). All combinations were diluted using ECBM without supplements and with 1% FBS. Images were taken every 10 min for 23 h using the Personal Automated Lab Assistant (Leica Microsystems). Areas of migration were measured using Fiji.

Scratch assays were performed using glass-bottom FluoroDishes seeded with 50,000 cells in 2 ml ECBM with 1% FBS at 5% CO_2_ and 37 °C. After 48 h, cells reached 90% confluence and were ready for the experiment, in which a sterile plastic 1,000 μl micropipette tip was used to scratch the cell monolayer to create a wound of around 1,000 μm. Next, the cell monolayer was gently washed with ECBM with 1% FBS to remove dead cell debris. To use the nucleus for tracking, PECs were stained with 80 nM Hoechst 33342 (Thermo Fisher Scientific) for 20 min at 5% CO_2_ and 37 °C and washed once with Dulbecco’s PBS (Thermo Fisher Scientific, 14190094). Afterwards, 2 ml fresh ECBM with 1% FBS was added for image acquisition. Time-lapse imaging was performed using a Leica DMi8 M/C/A inverted microscope equipped with ×10 Plan Apo objective (Leica Microsystems). Images at both sides of the wound were acquired every 5 min with an ORCA-Flash4.0 digital camera (Hamamatsu Photonics) using MetaMorph (v.7.10.3.279) software (Molecular Devices). To visualize the wound, adjacent positions were stitched using the Stitching plugin from Fiji ImageJ. Tracking of the first 8 h of migration was performed with the TrackMate plugin from Fiji (v7.10.2) and custom-made scripts^62^. Mean square displacement was calculated using the CelltrackR package^63^.

### Transmission electron microscopy

For electron microscopy with immunogold labelling, kidneys were removed, cut into 2-mm-thick razor blade sections and immersion-fixed in freshly prepared 4% paraformaldehyde for 24 h at 4 °C. The samples were then resliced into 50-µm-thick sections using a vibratome. Vibratome sections were incubated with the primary antibody against TRPC6 (rabbit, final concentration 1:200). After washing and overnight incubation at 4 °C with the secondary antibody, goat anti-rabbit 1:100 (Nanoprobes), sections were silver enhanced with HQ silver (Nanoprobes) for 8 min in the dark at 4 °C, washed in 0.1 M phosphate buffer, treated with OsO_4_ (0.5% for 45 min at room temperature) and stained with uranyl acetate (1% w/v in 70% v/v ethanol, 30 min at room temperature). After dehydration, sections were embedded in epoxy resin, Durcupan ACM (Sigma-Aldrich). Next, 50-nm ultrathin sections were cut using an UC6 ultramicrotome (Leica Microsystems) and analysed using an 80 kV Zeiss Leo 910 transmission electron microscope.

### Imaging mass cytometry

Tissue sections were dewaxed in xylene and rehydrated, followed by staining using a standard protocol for immunohistochemistry according to the protocol by Fluidigm. Nuclei were labelled with iridium, and TRPC6 antibody (Abcam, ab105845) was coupled to 174Yb heavy metal. Data acquisition was performed on a Helios time-of-flight mass cytometer (CyTOF) coupled to a hyperion imaging system (Fluidigm). Areas for ablation were selected on the basis of haematoxylin and eosin staining performed on an adjacent slide. All data were collected using the commercial Fluidigm CyTOF software (v.01).

### Pathological examination of crescentic nephritis

Images were evaluated by two expert pathologists in a blinded fashion to define disease states as either control, acute or crescentic phase. Next, multiple metrics were also calculated in a blinded fashion, including tubular injury score (0–3+), cell numbers per cross section, percentage of cellular crescents and percentage of tubular injury.

### Bulk RNA-sequencing sample preparation and analysis

Glomeruli were isolated at day 4 after NTS treatment and in control groups after kidney perfusion with Dynabeads (Invitrogen), preserved in RNAlater and stored at −80 °C until processing. For preparation of nuclei, nuclei were extracted from the isolated glomeruli according to a modified protocol^64^. The nucleus suspension was incubated on the magnet to remove magnetic beads used for the isolation of the glomeruli. Nuclei were mixed with RLT buffer (Qiagen) and frozen at −80 °C. Total nuclei RNA was extracted using RNeasy Micro kits (Qiagen) according to the manufacturer’s recommendations.

Bulk RNA-sequencing data were processed using our previously published open-source Snakemake^65^ workflow for RNA-sequencing analysis with pytximport^66^. In brief, raw FASTQ files provided by the sequencing facility were assessed for quality with FastQC^67^, followed by trimming of adapter sequences and removal of low-quality reads with fastp^68^. Next, processed sequences were selectively aligned to a reference gentrome based on Ensembl GRCh38 (release 112)^69^ and transcript counts were quantified with Salmon^70^. We used pytximport to estimate gene counts from transcript abundances with counts_from_abundance set to length_scaled_tpm. Differentially expressed genes were identified using PyDESeq2 (ref. ^71^) with log_2_-transformed fold shrinkage. Genes were considered differentially expressed if their log_2_-transformed fold change value was greater than 0.5 or lower than −0.5 and their false-discovery-rate-adjusted *P* value was less than 0.01. The results from PyDESeq2 were used to infer transcription factor activity based on the CollecTRI^72^ gene regulatory network reference with decoupler-py^73^ univariate linear modelling.

### Python library for spatial proteomics

Commonly used functions for the analysis of PathoPlex imaging data were combined into the spatiomic Python package. spatiomic comprises different submodules that facilitate data loading, image registration, image preprocessing, dimensionality reduction, spatial analyses, neighbourhood graph construction and clustering. In this section, we describe the architecture of this software package and the general functionality it includes. Parameter choices and detailed workflows are described in subsequent sections. Both the computational analysis library and the code for all analyses are available online through GitHub and Zenodo, as detailed in the Code and Data availability sections.

spatiomic comes with support for multiple common microscopy imaging formats and flexibly supports AnnData^74^ objects, NumPy^75^ arrays and pandas DataFrames^76^. It uses cuml, cucim and cugraph from the RAPIDS ecosystem^21^ as well as cupy^77^ for GPU-accelerated computations, which enables analyses to scale to billions of data points with affordable hardware with a time requirement of just minutes to hours depending on dataset size. The library was extensively unit-tested, typed and documented. Documentation, including a full example notebook detailing how to apply spatiomic analyses to PathoPlex data, is available at: https://spatiomic.org/.

To enable modular composition of analyses, spatiomic encompasses several submodules as described below.

#### Data submodule

The data.read class offers a method for parsing common microscopy imaging formats such as .tiff, .lif and .czi files through readlif, tifffile and aicspylibczi bindings. A random subsample of imaging data can be obtained through the data.subsample class. The submodule further contains functionality to subset multichannel images to a specified list of channels and to export data to AnnData^74^ objects, which enables interoperability with the scverse^23^.

#### Process submodule

The process submodule offers common functions for preprocessing imaging data. This includes the clip class for channel-wise histogram clipping to either absolute values or percentiles, the zscore class for channel-wise *z* scoring and the normalize class for the channel-wise scaling of intensity values to a given range. It also includes the register class, which exposes different methods for image registration and registration evaluation.

#### Dimension submodule

Dimensionality reduction is an important step in many single-cell and spatial omics analyses. spatiomic provides classes that help reduce the dimensionality of both the channel dimension and the data point dimension. The former is possible through the integration of the dimension.pca, dimension.tsne and dimension.umap classes, which internally rely on the Python packages scikit-learn^78^, umap-learn^79^ and cuml^21^. The latter is achieved by incorporating the dimension.som class, which enables GPU-accelerated training of SOMs thanks to an XPySOM^22^ integration. To train SOMs with the Pearson correlation as distance metric, we extended XPySOM with a CuPy-based function, which is available from GitHub (https://github.com/complextissue/xpysom).

#### Neighbour submodule

The neighbour submodule exposes classes that enable the creation of *k*-nearest and shared nearest neighbour graphs, which facilitates the construction of similarity-based graphs for graph clustering and distance-based neighbourhood graphs for spatial analysis.

#### Cluster submodule

Clustering algorithms enable the unsupervised identification of similar (protein co-expression) patterns, which facilitates automatic partitioning of complex signals into biologically meaningful groups. spatiomic.cluster includes classes for GPU-accelerated clustering with the Leiden graph clustering algorithm^24^, *k*-means and hierarchical agglomerative clustering.

#### Spatial submodule

The spatial submodule incorporates functions for the explorative analysis of spatial distribution patterns in both immunofluorescence and clustered images, including global and local univariate and bivariate measures of spatial distribution based on PySAL^80^. It further includes code for efficient join count quantification and spatial vicinity graph construction and interoperability tools for use together with PySAL^80^, thereby ensuring compatibility with a wide range of spatial statistics applications^81^.

#### Tool submodule

The tool submodule contains utility functions for additional evaluation or analysis. It enables quantification of cluster abundance and identification of significantly differentially expressed clusters, calculation of mean immunofluorescence marker intensities per cluster and identification of cluster-defining immunofluorescence markers.

#### Plot submodule

spatiomic includes plotting functions based on matplotlib^82^ and seaborn^83^ that facilitate visualizing common plots, for example, image registration metrics, SOM training-quality metrics, cluster projections, spatial adjacency graphs as well as cluster contributor histograms and volcano plots.

#### System and time requirements

spatiomic is designed to be flexible and adaptable to the scale of multiplexed imaging data and available computing resources. The only mandatory system requirement is Python (v.3.10) or higher. Although many individual functions run in seconds, and a complete exemplary workflow (excluding data download) can be completed in less than 3 min on a standard personal laptop, spatiomic substantially benefits from CUDA-enabled GPUs compatible with the RAPIDS ecosystem for larger datasets. At the scale of analysis presented in this paper, three specific steps took several hours to complete on a single GPU. First, image registration time scales linearly with the number of ROIs acquired and imaging cycles. Second, the time for SOM training scales linearly with the training sample size, the number of training iterations and the number of SOM nodes. Finally, the transfer of cluster labels from the trained SOM to all images in the dataset scales linearly with the number of acquired ROIs and the number of SOM nodes. Therefore, users are encouraged to evaluate and test parameter choices to optimize performance for their specific experimental design. For example, smaller, more homogeneous datasets may benefit from smaller training subsamples and SOM sizes, which will result in faster analysis. Moreover, users should select hardware that is appropriate for their analytical needs and desired turnaround time.

### Image registration and autofluorescence correction

Image registration is the first step of every computational analysis of PathoPlex images. Given the cyclical nature of image acquisition and imperfect repositioning of standard microscopy systems, aligning signals from all imaging cycles to a common reference is a prerequisite for joint analyses.

We propose two different ways of aligning iteratively acquired immunofluorescence images: on the basis of either a nuclear or a pan-protein stain as a registration reference. Although nuclear markers are widely used for registration, a pan-protein marker enables the alignment of images and facilitates the delineation of the portion of the image covered by tissue, thereby lowering the computational burden for downstream processing and reducing the cluster annotation time by masking the empty background. To combat autofluorescence due to red blood cells (RBCs) in samples with abundant RBCs, we further used a RBC marker to specifically remove this source of noise. Detailed parameter choices and method descriptions are provided for the respective experimental datasets in the subsequent subsections. Differences between datasets, such as the use of NHS-E for foreground segmentation and the use of glycophorin A for erythrocyte segmentation in selected datasets, represent the iterative improvement of PathoPlex, with earlier experiments informing strategies for further scaling in subsequent experiments. In particular, the preprocessing steps described in this section refer only to the transformation of nuclear staining or NHS-E for registration purposes and do not indicate the transformation of marker intensities as used for further analysis, unless indicated otherwise.

#### Mouse CGN experiment

To align cycles from the CGN mouse dataset (Fig. 2), nuclear reference images (DAPI in cycles 1–17, DRAQ5 in cycles 18–42) were clipped image-wise to the range between the 1st and 99.9th intensity percentiles. Intensity values were *z* scored and normalized to the range 0–1. Gaussian blur with a sigma of 1 was then applied to the nuclear reference images to reduce noise and to improve comparability across cycles, followed by histogram matching of all subsequent images to the histogram of the reference image from the first cycle. These processed nuclear images were then aligned to the processed refence nuclear image obtained in the first imaging cycle. To this end, all intensity values below the 70th intensity percentile were set to 0 in both the reference nuclear staining and the staining to be aligned to increase the contrast between the nuclei (constituting less than 30% of the imaged pixels in all images) and the unstained background, followed by *x* and *y* offset detection between images with the phase_cross_correlation function of the Python package scikit-image^84^, as included in spatiomic.process.register. All channels were projected onto the first cycle on the basis of the detected offset. Finally, the maximum offset detected between any two cycles was subtracted from all sides of all registered images, which resulted in square-sized and equally sized images.

#### Human advanced DKD experiment

For the study of specimens from individuals with advanced DKD and from individuals without diabetes (Fig. 3), all images were first corrected for camera distortion using the remap function of OpenCV^85^ by linear interpolation based on a reference image of a micrometre-scale microgrid captured with the respective microscope objective. Histogram matching of the nuclear channel for each cycle to the reference nuclear channel of the first cycle was used to increase the similarity between nuclear images. Following that, the phase cross-correlation offset detection, as implemented in spatiomic.process.register.get_shift, was used to align all images to the first imaging cycle on the basis of the *x* and *y* offsets between the nuclear (DRAQ5) channels. For each field of view (FOV), the overlap across all cycles was calculated and the images were cropped to the respective overlap area.

#### Human early diabetes treatment experiment

This experiment (Fig. 4) leveraged NHS-E, a pan-protein stain, as a registration reference. To facilitate registration and further image processing, we applied local mean downscaling with a 2 × 2 pixel kernel to all imaging data. We then clipped the NHS-E histogram to the range between the 1st and 95th percentiles and normalized the NHS-E intensity to the range 0–1. After NHS-E preprocessing, Gaussian blurring with a sigma of 1, thresholding at the 70th percentile and phase correlation-based offset detection with an upsampling factor of 5 were applied to identify the *x* and *y* offset between NHS-E images from all cycles, using the first imaging cycle as the reference. After correcting for this offset, SIFT-based homography detection^86^ was performed and images from subsequent cycles were warped to match the reference from the first cycle. Pre-registration and post-registration structural similarity index metrics (SSIMs) were evaluated for all registration pairs. Manual registration checks were performed for images with the following criteria: the SSIM decreased following registration; the SSIM after registration was lower than 0.3; any offset was greater than 300 pixels; or the SSIM after registration was lower than 0.8 and the SSIM improvement lower than 0.05. To account for RBC-derived autofluorescence, glycophorin A intensities across all images were clipped to the range between the 90th and 99.5th percentiles, and Otsu thresholding was used to binarize signals and to create RBC masks. Masks were smoothed through the removal of small objects <72 pixels, two iterations of binary dilation and hole filling. To remove signals from empty background without tissue, processed NHS-E signals were combined across all cycles by minimum projection (thus excluding areas with lifting at any point) for each image and Otsu thresholding was used to binarize signals. All images were then restricted to the area covered by the NHS-E mask but not included in the RBC mask. Autofluorescence correction was performed using secondary-antibody cycles acquired repeatedly throughout the experiment. For each primary-antibody imaging cycle, tissue autofluorescence was estimated by interpolating the signals from the nearest preceding and subsequent secondary-only cycles at the same wavelength, thereby accounting for minor fluctuations in autofluorescence. This estimated autofluorescence was then subtracted from the corresponding channels with lower intensity clipping at zero.

#### Other immunofluorescence samples

To achieve reliable and accurate results, we used an iterative registration framework called Elastix^87^ and a Python wrapper package called PyElastix^88^. To improve contrast and to mitigate the effects of varying signal strength across cycles, we applied histogram equalization to the nuclear channels. We also reduced the computational load by rescaling the images by a factor of 0.25. The first cycle was established as a reference, and subsequent cycles were aligned to it using normalized correlation as the optimization metric. The registration process involved iterative steps at six different resolution levels, with 1,000 iterations per level. We used the rigid Euler transform to account for *x* and *y* offsets as well as rotation. Further preprocessing was performed for brain samples with reduced contrast by truncating pixel intensity values to high percentiles. This approach focused on the sparsely available nuclei. All registrations were manually verified, and if individual registrations were unsuccessful, minor adjustments were made to the downsampling factor, spatial sample number or iteration number until satisfactory registration was achieved to maximize image inclusion.

#### Alignment of PAS staining to immunofluorescence images

Owing to differences in size and lens characteristics between the immunofluorescence and PAS images, additional processing steps were required to align the two modalities. First, lens-specific calibration matrices were used to remove optical distortions as described above for the human advanced DKD experiment. Next, the PAS images were rescaled to match the physical pixel resolution of the immunofluorescence images. As only greyscale images were used for registration, the PAS images were converted from RGB to greyscale using the OpenCV^85^ Python library. For the greyscale version of the immunofluorescence image stack, three structural marker channels (DRAQ5, LTL and collagen IV) were selected and combined into a single channel using weighted addition. To enhance visual similarity between the greyscale PAS and immunofluorescence variants, the PAS image was inverted, and histogram equalization was applied to both the PAS and immunofluorescence images. To establish an initial alignment for registration of the immunofluorescence onto the PAS image, the PAS images were centrally cropped to match the smaller size of the immunofluorescence images (3,000 × 4,000 versus 2,048 × 2,048 pixels, respectively). Once the *x* and *y* offsets in this subregion were determined by the registration algorithm, the original multichannel immunofluorescence images were transformed accordingly. The full-size RGB PAS images were also cropped to the size of the transformed immunofluorescence images to facilitate overlaying of the registration results.

#### Suitability of registration reference markers

In a subsequent investigation, our goal was to evaluate the suitability of DAPI, DRAQ5 and NHS-E as registration reference labels. To achieve this goal, we acquired images of all three marker channels for each imaging spot in each cycle. For each image requiring registration, transformations based on each of the three markers were independently computed. The corresponding DRAQ5 channel of each image was adjusted to account for the detected transformation, and the structural similarity index measure was computed by comparing it to the DRAQ5 channel of the reference cycle. This approach ensured that the different registration references could be compared using DRAQ5 as a quality metric, which consistently provided reliable and high-contrast images. All registrations were manually reviewed, and in instances where registration was not successful, minor adjustments were made to the downsampling factor, the spatial sample number or the iteration number until a satisfactory registration was achieved. This process ensured that no image had to be discarded and that high-quality alignments were obtained.

### Quality control of automated elution

Complete elution of bound antibodies between cycles is key to the cyclic acquisition of images. To quantify elution efficiency, secondary-antibody-only staining cycles were acquired throughout the experiments to establish a baseline autofluorescence profile. For each primary-antibody staining, the 50th and 99.95th intensity percentiles were quantified and compared with the secondary-antibody-only cycles acquired at the same wavelength using secondary antibodies directed against the host species of the primary antibody. For instances when at least one value was equal to or lower than any intensity from the secondary-antibody-only cycles (as may be the case for non-abundant markers, for example, markers for rare immune cells or phosphorylation states), the images were manually re-evaluated to include specific staining.

### Generation and interpretation of protein co-expression clusters

Protein co-expression clusters are the standard output of PathoPlex analyses with spatiomic, which captures specific co-expression patterns of different proteins at the pixel level. These patterns are jointly identified for all imaging data from each respective PathoPlex experiment, which results in a consistent clustering across all samples and facilitates comparisons of spatial expression or co-expression of immunofluorescence markers. Identification of these pixel-level clusters is a multistep process that consists of weighted random subsampling of the data to ensure equal representations of all desired variables, signal preprocessing, training of a SOM to identify representative co-expression signals and finally similarity graph-based clustering. Once clusters were identified, their constituting signals were compared to all other clusters and projected in space to infer the biological processes they represent. Last, cluster abundance was quantified and compared across conditions to delineate regulated signals. We applied this overall concept to each experimental dataset with parameters specified as described in the subsequent subsections.

#### Weighted random subsampling

To limit bias due to different data sizes between samples and to reduce the computational burden of pixel-based clustering, a weighted subsample of approximately 5 million (10 million for the early human diabetes dataset) random pixel positions per imaging plate were sampled. For each imaging plate (*n* = 1 for all experiments, except for the human advanced DKD experiment, for which *n* = 2 imaging plates were used), the number of subsampled pixel positions was equally distributed across all disease states, and for each disease state, all samples were equally weighted. Finally, each FOV of a given sample was given the same weight in the subsample. For the early human diabetes treatment dataset, the subsample also considered equal numbers of pixels from periglomerular, glomerular and tubular images.

#### Histogram clipping and normalization

Immunofluorescence markers differ in intensity range and abundance, which therefore requires preprocessing steps to improve comparability. On the basis of weighted random subsamples, histogram clipping and range normalization classes contained in the spatiomic.process submodule were fitted. After channel-wise fitting of the classes on the random weighted subsample, all channels of all images of each respective dataset were transformed according to the established clipping limits and normalization and scaling settings. For mouse samples and the advanced DKD experiment, histogram clipping was performed based on the 50th (lower) and 99.7th (upper) percentiles for each respective marker in the subsample. Owing to the extensive marker panel for the larger early human diabetes treatment experiment, coupled with the increased sensitivity of the confocal microscopy system used for this dataset, histogram clipping was performed based on absolute intensity thresholds established by human expert annotation in a condition-blind fashion, with each marker evaluated on random patches extracted from random images, followed by normalization of the clipped images.

#### SOM fitting

Pixel-based clustering was used to isolate groups of similar immunofluorescence marker signals (clusters), which formed the basis of all downstream analyses. The first step towards this clustering is the fitting of SOMs to the dataset to reduce data-point dimensionality and to ensure computational feasibility of graph clustering as well as to improve representation of signals that are relatively rare in the training data. For each imaging plate (*n* = 2 for the human advanced diabetes experiment, *n* = 1 for all other datasets), a SOM was trained on the corresponding weighted random subsample. The SOM was initialized with a grid size of 500 × 500 nodes (400 × 400 for the early diabetes experiment) and used the Euclidean distance metric for the mouse CGN dataset and the advanced human diabetes dataset with the cosine similarity metric used for the early human diabetes experiment. The training process used spatiomic.dimension.som. For human samples, a final learning rate of 10^−4^ and a final Gaussian neighbourhood sigma of 10^−3^ (3 × 10^−3^ for the early human diabetes dataset) were used. For the mouse samples, the default settings for the learning rate and neighbourhood size were used. The training process was repeated for 50 iterations for all datasets.

#### Graph clustering and batch integration

On the basis of the representation of the signals contained in each experiment as provided by the respective SOM nodes, we used Leiden graph clustering to identify clusters of protein co-expression patterns and applied the clusters to all pixels from all images for each dataset. First, the similarity-based neighbourhood graph of SOM nodes was built using spatiomic.neighbor.knn_graph using cosine (early diabetes treatment experiment) or Euclidean distance (all other experiments). When image acquisition was performed using multiple plates (Fig. 3), the neighbourhood graph construction step was modified to use an adaptation of the batch-balanced *k*-nearest neighbours^89^ algorithm, implemented in spatiomic.neighbor.knn_graph. A neighbour count of *k* = 40 for each respective plate was used. When only one imaging plate was used (all other experiments), a neighbour count of *k* = 40 (*k* = 50 for the early human diabetes experiment) was used without any batch integration. After graph construction, we used the Leiden^24^ graph clustering algorithm to identify clusters of similar protein co-expression patterns. A Leiden resolution of 2.5 and an iteration count of 10,000 were used for the advanced DKD experiment, a resolution of 2.0 and an iteration count of 1,000 for the early human diabetes treatment experiment and a Leiden resolution of 1.0 and an iteration count of 10,000 for the mouse CGN experiment samples.

#### Cluster identity

To assign biological identities to protein co-expression clusters derived from PathoPlex output, we used a two-step approach that combined statistical overrepresentation with spatial distribution analysis and signal interpretation by human experts. Owing to computational constraints, statistical analyses were performed on subsampled data (used with the early diabetes experiment, with 500,000 pixels randomly selected from the weighted random subsample) or the representative SOM nodes (all other datasets). For each cluster, we calculated the mean normalized intensity and the log_2_-transformed fold change of marker intensity (relative to all pixels or nodes not assigned to that cluster), which reflected the signal strength and cluster specificity, respectively. Significance was assessed using a two-tailed *t*-test with Benjamini–Hochberg (early diabetes dataset) or Holm–Šidák (all other datasets) correction for multiple testing (implemented in spatiomic.tool.get_stats). Dominant markers in each cluster (termed high contributors) were identified by ranking markers based on the product of their mean intensity and log_2_-transformed fold change, and retaining only those with mean normalized intensity ≥ 0.2, adjusted *P* < 0.05 and log_2_-transformed fold change ≥ 1. Intensity histograms further visualized marker distribution patterns in each cluster for all markers and were also considered for cluster interpretation. Individual clusters were spatially projected onto the corresponding immunofluorescence and—where available—PAS-stained images. Human experts then visually validated and refined cluster identities by assessing their spatial distribution, considering morphological structures and correlations with immunofluorescence signals and integrating contextual biological knowledge.

#### Selection of specific foreground clusters

As multiplexed imaging data from Figs. 2 and 3 did not include a pan-protein marker in their panel, no foreground segmentation was performed before pixel-level clustering, which resulted in multiple clusters that corresponded to empty background areas. To account for this limitation (largely circumvented by NHS-E and a larger antibody panel in Fig. 4), we restricted extended analyses (but not differential cluster abundance analyses) to specific foreground clusters only or we treated all background clusters as a single cluster. For Fig. 4, we similarly assessed the specificity of all clusters and quantified their relative frequency, focusing visualizations and biological assessment to clusters deemed to represent specific signal (excluding minor imaging artefacts, autofluorescence signals and imperfect foreground segmentation) and accounting for >0.1% of foreground pixels. The specificity of clusters, defined as the extent to which clusters correspond to clear biological processes and/or structures, both at the spatial and at the molecular level, was assessed individually by a panel of three experts. In controversial cases, the experts presented their arguments until a unanimous decision for exclusion was reached. If arguments for exclusion were not unanimously accepted, the cluster was not excluded.

#### Cluster abundance and differential abundance analysis

Cluster abundances were analysed by quantifying the number of pixels assigned to each cluster for each field of view in a dataset using spatiomic.tool.count_clusters. The abundances were normalized to a range of 0–1 to facilitate comparison and interpretation across FOVs. For the mouse CGN and the advanced diabetes experiment, these normalized abundances were further aggregated to obtain the mean cluster abundance per field of view for each mouse or patient. Differential abundance analysis was performed by quantifying the log_2_-transformed fold change in cluster abundance between different experimental and clinical conditions by performing a two-sided *t*-test. To account for the higher number of unique clusters and larger sample sizes, *P* values for the advanced DKD and the early diabetes treatment experiments were corrected for multiple testing with Benjamini–Hochberg false discovery rate adjustment. Statistical testing used the spatiomic.tool.get_stats function. Results were visualized with spatiomic.plot.volcano for all datasets. For visualization purposes, the volcano plots were restricted to feature clusters determined to represent specific foreground signal. Moreover, to assess the impact of batch integration, quality control was performed by comparing differences in log_2_-transformed fold changes in inter-group cluster abundances between the imaging plates (Supplementary Fig. 12).

### Extended analyses based on pixel-level clusters

Based on our foundational pixel-level protein co-expression clusters, we used multiple downstream applications to connect the output from PathoPlex with community resources and public knowledge bases, showcasing how pixel-level data can be aggregated at different levels to derive information across multiple biological scales.

#### Biclustering

UnPaSt^42^ is a biclustering method initially developed for unsupervised patient stratification based on omics data. UnPaSt identifies differentially expressed biclusters in a two-dimensional matrix with samples (for example, images or patients) in columns and features (for example, clusters) in rows. A differentially expressed bicluster is a submatrix consisting of samples and features such that these features are overexpressed or underexpressed in these samples compared with all other samples in the input data matrix. We applied UnPaSt with binarization *P* value threshold of *P* = 0.05, direction = ‘both’ (to identify biclusters consisting of both upregulated and downregulated clusters) and all other parameters set to default to image-level and patient-level cluster intensities. As UnPaSt is not deterministic, consensus biclusters were built on the basis of results of ten independent runs.

#### Druggability profiling

To evaluate the druggability of the molecular signature of advanced DKD and to identify potential therapeutics, we combined drug–protein interaction data from the CTD^44^ with STRING^90^ protein–protein interaction data. Initially, we manually identified AIFM1, TRPC6, CALR, HSPA5, ITGB1 and CTNNB1 as possible targets involved in the altered signalling cascades revealed by PathoPlex based on the significantly differentially expressed clusters and their molecular composition. Next, we used STRING data for *Homo sapiens* to link every potential target to a broader network of interacting proteins. We only considered direct interactions with a confidence score exceeding 0.75. In a subsequent step, CTD chemical–gene interaction data were used to extract possible therapeutics that affect proteins in our target networks. This search was limited to compound–protein interactions described in mice (*Mus musculus*), rats (*Rattus norvegicus*) or humans. Only interactions that did not involve co-treatment and did not affect the reaction of another externally administered compound were included. Our goal was to determine the impact of existing pharmacological treatments on the extended protein networks and to explore the potential for repurposing drugs authorized for other indications; therefore, we further filtered the results, preserving only entries for which the chemical name had a matching entry in the European Medicines Agency’s list of authorized agents (date of consultation: 23 August 2023). In a second step, to further assess the impact of current antidiabetic drugs, results from all protein networks were combined and filtered for compounds containing ‘glutid’, ‘gliflozin’, ‘glitazon’, ‘gliptin’, ‘metformin’, ‘pril’ or ‘sartan’.

#### Pixel cluster-assisted cell-level metaclustering

Although pixel clusters provide valuable subcellular and extracellular information, they can also be used to inform existing cell-level clustering workflows. To quantify the co-occurrence of pixel-level clusters within cell-level metaclusters, we first applied the Cellpose^39^ segmentation model using the parameters model_type = “nuclei” and diameter = 30 to the pre-processed DRAQ5 channel of each image. Statistical testing of nucleus counts per image was performed using statannotations^91^ with a two-sided nonparametric Mann–Whitney *U*-test, comparing diabetes and control samples. Centroids of all identified nuclei were then expanded radially by the smaller of either 5 µm or 50% of the shortest Delaunay triangulation edge length to approximate cell areas. Within these estimated areas, the relative abundances of pixel-level clusters were calculated to produce feature vectors for each cell. To identify cell-level metaclusters, a *k*-nearest neighbour graph was constructed using spatiomic.neighbor.knn_graph with neighbor_count = 50 followed by Leiden^24^ clustering with a resolution of 1.0. Condition-specificity of meta-clusters was confirmed by averaging their relative abundances across images at the patient level. Within each cell-level metacluster, the fraction of pixels corresponding to each pixel-level cluster was quantified, which provided a quantification of the cell-level co-occurrence patterns of pixel clusters. Differences in normalized metacluster abundance at the patient-level between conditions were visualized through a two-component principal component analysis using spatiomic.dimension.pca with default parameters and significance was established through a Mann–Whitney *U*-test with Bonferroni correction.

#### Pseudotime analysis

PILOT^41^ is a previously published, multiscale, unsupervised method that uses optimal transport to compute the distance between data points and infer a disease trajectory. First, we used the normalized abundance of the clusters as the cluster proportions for each FOV from all samples. Next, we computed the distances between the clusters as a cost matrix. In the subsequent step, we used the cluster proportions and the cost matrix to compute the Wasserstein distance^92^ (W1) between the data points. Then, we obtained the trajectory of the disease by applying the diffusion map^93^ to the distance matrix of the samples. Finally, we used the assigned pseudotimes of the data points to reveal the changes in the proteins or clusters. In summary, PILOT uses stepwise nonlinear models to determine significantly changing proteins or clusters across the disease trajectory.

#### Cluster join counts analysis

The immediate spatial neighbourhood analysis was based on join counts between unique clusters that were used to create an adjacency graph using spatiomic.spatial.vicinity_composition. First, for each pixel position, the eight surrounding pixel positions (Chebyshev distance of 1 pixel; that is, first-order queen neighbourhood) were examined to count the instances of nearby clusters. This process was applied to all pixels in all images using a vectorized approach. The cluster counts were then aggregated at the dataset level for images of the same disease condition. Next, connections between identical clusters were discarded, and the remaining connections were normalized to a range of 0–1. Non-foreground clusters were discarded. The resulting adjacency matrix was used to construct a directed graph for each condition, with the graph representing the relationships between clusters. Although the entire adjacency matrix was quantified and evaluated, a neighbourhood cluster abundance of 7.5% was established as minimum value to be included in the graph plot for visualization purposes, focusing the visualization on the most common adjacencies. The graph layout was calculated using the software packages Graphviz^94^ and NetworkX^95^.

#### Condition-specific structural patterns

Condition-specific structural patterns were identified using MISTy^40^ (mistyR v.1.6.1). To that end, image data (112 images from advanced DKD samples and 310 from control samples) were aggregated at two different resolutions by summing the cluster counts in bins with a side length of 10 μm (62 × 62 pixels). Clusters capturing empty background, unstained tissue parts and nonspecific signals were collapsed into a single background cluster. To account for truncated bins at the edges of slides, we transformed the counts into proportions. We used these cluster proportions per bin as an intrinsic representation of the structure in a bin (MISTy intraview). To capture the broader tissue structure, we constructed the paraview by summing up the cluster proportions of the 20 nearest neighbours using family = “constant”, l = 20. To construct the paraview for the high-resolution aggregation, we computed the weighted sum of the cluster proportions of the 80 nearest neighbours using a Gaussian kernel with a bandwidth of 2.5 μm (corresponding to 15 pixels) (family = “gaussian”, l = 2.5, nn = 80). With these view compositions per aggregation, a MISTy model was independently trained for each sample. The MISTy models identified significant structural patterns in the different spatial contexts by associating the proportion of pixels belonging to each cluster in each spatial context to the target proportions in the intraview. MISTy can learn both simple linear relationships (for example, cluster X has a higher proportion if cluster Y has a lower proportion) and complex nonlinear relationships. By combining the predictions from the intraview and paraview for each cluster, MISTy enabled us to disentangle whether the prediction for a given cluster improves, and to what extent, when taking different spatial contexts into account. To compare the MISTy importance scores between conditions, we first computed the mean results per sample due to differing numbers of imaged FOVs. We then aggregated the MISTy results per patient and finally per condition (advanced DKD and controls). For each level of aggregation, group and view, we generated a graph representing the inferred relationships between clusters. In each graph, the nodes represent the clusters and the edges between the clusters were weighted by the importance scores inferred by the MISTy model (thresholded to conserve only significant relationships with importance > 1.0). The graph layout was calculated using Graphviz^94^ and NetworkX^95^.

### Single-cell and single-nucleus RNA sequencing

To contrast our findings at the protein-level with transcriptomic data and to establish a bridge to proposed treatments of DKD, we leveraged two public RNA-sequencing datasets with pharmacological intervention or different treatment data, covering rodent and human samples.

#### Processing single-cell RNA-sequencing data

Data were downloaded from the Gene Expression Omnibus (accession: GSE220939). Files from individual patients were converted to AnnData^74^ objects, and information on the diabetes and SGLT2i treatment status of each patient was added. To remove ambient RNA contamination, Cellbender^96^ (v.0.3) training was performed on each sample individually with a training fraction of 0.5 for 100 epochs. Observations with a Cellbender cell probability ≤ 0.5 were discarded. Next, all observations were combined into a single AnnData object, quality-control metrics were quantified and cell clustering was performed using the Python package scanpy^97^. As part of this step, mitochondrial, ribosomal and haemoglobin genes were identified, and quality control metrics were calculated using the calculate_qc_metrics function. Barcodes containing ≥50% mitochondrial RNA, ≥20% rRNA or ≥5% haemoglobin genes were discarded. Barcodes with reads for less than 500 or more than 5,000 genes were discarded to correct for doublets, as were genes detected in at most 4 observations. The total number of reads per cell was normalized and the counts were log1p transformed. To reduce dimensionality, the first 50 principal components were computed using scanpy’s pca method with the arpack singular value decomposition solver. As inter-individual batch effects were present, Harmony^98^ data integration was performed with standard parameters, using the harmonypy^99^ implementation available through the external module of scanpy. On the basis of the adjusted principal components, the 50 nearest neighbours of each cell were identified using the neighbours function of scanpy^97^, which was configured to use the Pearson correlation similarity metric. Graph clustering was performed using the Leiden algorithm^72^ with a resolution of 0.5. On the basis of cell-identity genes (adapted from a previous study^46^ and PanglaoDb^100^), clusters were manually annotated to represent different cell types. Cell types and transcripts related to the pathways altered at the protein level were chosen, and gene counts were visualized using the dotplot function of scanpy^97^. Statistics per cell type of interest were calculated on the depth-normalized gene counts using the get_stats function of spatiomic, configured to revert log1p transformation. Nonparametric independent Wilcoxon rank-sum testing was performed using the ranksum function of scipy^101^ with Benjamini–Hochberg correction.

#### Processing single-nucleus RNA-sequencing data

Preprocessed data were downloaded from the Gene Expression Omnibus (accession: GSE209821). Gene counts were read into R using Seurat and converted to AnnData^74^ with SeuratData and SeuratDisk. In the next step, the AnnData object was read using the scanpy^97^ Python library, and provided metadata were combined with the gene counts. The provided counts had already been filtered for quality-control metrics and cluster labels from a previous study were provided^47^, thus, only depth normalization to the median read depth was performed. Observations were further filtered to only retain proximal tubule cells from controls, untreated mice or mice treated with soluble guanylate cyclase activators. Differential expression statistics of genes corresponding to defining markers of clusters upregulated in the proximal tubular compartment in the advanced DKD PathoPlex experiment were calculated based on depth-normalized gene counts using the spatiomic.tool.get_stats function, configured to run nonparametric independent Wilcoxon rank-sum testing using the ranksum function of scipy^101^ with Holm–Šidák correction.

### Signal intensity of TRPC6 and AIFM1 in *Btbr*^*ob/ob*^ mice

To further evaluate the expression of TRPC6 and AIFM1 in the proximal tubule of kidneys with metabolic damage, we assessed their expression in *Btbr*^*ob/ob*^ mice and control mice at 12 and 24 weeks of age. Immunofluorescence was performed on 5 FOVs per sample, with *n* = 3 samples per group and time point, which produced a total of *n* = 12 samples and 60 FOVs. The proximal tubule area was segmented by thresholding the LTL channel to an intensity greater than 1,500, followed by filling holes smaller than 1,000 pixels and removing objects smaller than 20,000 pixels. After proximal tubule segmentation, a random subsample of 50,000 pixel positions was selected from the proximal tubule area of each FOV. These subsamples were combined for each unique condition and time point. Changes in TRPC6 and AIFM1 expression were visualized through histograms of marker intensities, grouped by time point and condition. Statistical analysis was performed using the statsannotations^91^ Python package, for which the subsampled intensities served as input, with the corresponding condition as the label for each intensity. An independent *t*-test based on the statannotations^91^ Python package was used to assess statistical differences in TRPC6 expression between conditions at each time point.

### General statistical analysis

Statistical analyses, including cluster composition, differential cluster abundance and differential gene expression, were performed using spatiomic*.*tool.get_stats and statannotations. These tools internally rely on scipy^101^ and statsmodels^102^ and were used to perform either Benjamini-Hochberg or Holm–Šidák-corrected two-tailed *t*-tests (for cluster composition and differential abundance analysis), Mann–Whitney *U*-tests (for differences in nucleus counts and cell-level metacluster abundance) or nonparametric Wilcoxon rank-sum tests (for differential gene expression from single-cell and single-nucleus RNA-sequencing data). Bulk RNA-sequencing differential gene expression analysis was performed using PyDESeq2, combining single-factor analysis using Wald tests with log_2_-transformed fold change shrinkage with approximate posterior estimation generalized linear models^103^. Detailed descriptions of these tests, including information on input data and additional filters, are provided throughout the Methods. All other statistical analyses, including the quantification of changes in cell migration, clinical parameters (for example, proteinuria or eGFR), sample-specific variables (such as age), PEC activation, injury patterns in CGN and principal component analysis, were performed using GraphPad Prism (v.9). Violin plots report median and interquartile values. Significance was evaluated using the unpaired *t*-tests with Welch’s correction comparing two continuous variables, a paired *t*-test for before and after settings, and the Brown–Forsythe, Welch ANOVA and Dunnett’s tests when comparing three continuous variables. Correlation analyses were performed using Spearman rank coefficients. Principal components were selected on the basis of the percentage of total explained variance using normalized cluster abundances at the image-level or the patient-level as input. Statistical significance was defined as *P* < 0.05 for all analyses, with a threshold of *P* < 0.01 applied for specific cases as outlined throughout the methods.

### Reporting summary

Further information on research design is available in the Nature Portfolio Reporting Summary linked to this article.

## Online content

Any methods, additional references, Nature Portfolio reporting summaries, source data, extended data, supplementary information, acknowledgements, peer review information; details of author contributions and competing interests; and statements of data and code availability are available at 10.1038/s41586-025-09225-2.

## Supplementary information

Supplementary Information Supplementary Figs. 1–12, Supplementary Data 1 and 2 and Supplementary Video legend.

Reporting Summary

Supplementary Tables Supplementary Tables 1–9.

Supplementary Video 1 3D printing-based liquid-handling solution. This video shows the use of a 3D printer with custom 3D-printed cartridges and imaging chambers to perform automated liquid handling following the PathoPlex protocol.

## Source data

Source Data Fig. 2

Source Data Fig. 3

Source Data Fig. 4

## Data Availability

The bulk RNA-sequencing data from NTS-treated mice have been deposited into the Hamburg University Research Data Repository (10.25592/uhhfdm.17394). The public single-cell and single-nucleus RNA-sequencing datasets used in this study are available through Gene Expression Omnibus with accessions GSE220939 and GSE209821. PathoPlex animal multiplexed imaging data are available from Zenodo (10.5281/zenodo.15212140)^104^. For human data, as a patient re-identification key is retained internally for scientific continuity of ongoing projects, and historical versions of data containing patient identifiers persist in secure institutional servers and physical laboratory records, the raw microscopy data cannot be fully anonymized and therefore cannot be deposited in a public repository in accordance with General Data Protection Regulation. Raw data can be made available upon reasonable request and subject to a material and data user agreement that ensures appropriate safeguards for data protection and privacy in compliance with General Data Protection Regulation. The senior corresponding author will respond to data requests, aiming to answer within 72 h, and provide data up to 1 month after the material and data user agreement has been signed by both parties. Source data are provided with this paper.

## Code availability

The code for the 3D printer-based liquid-handling system and the STL files for 3D printing, the spatiomic library code and the code for the analysis of all datasets are available from Zenodo (10.5281/zenodo.15211354)^105^. The latest development version of spatiomic can be accessed from GitHub (https://github.com/complextissue/spatiomic). The documentation for spatiomic is available at: https://spatiomic.complextissue.com and includes example workflows for common analyses, including code to download example data. The following resources are available from GitHub: bulk RNA-sequencing workflow (https://github.com/complextissue/snakemake-bulk-rna-seq-workflow/); MistyR (https://github.com/saezlab/mistyR/); PILOT (https://github.com/CostaLab/PILOT/); UnPaSt (https://github.com/ozolotareva/UnPaSt) and the custom XPySOM adaptation that includes support for the Pearson correlation distance metric (https://github.com/complextissue/xpysom).

## References

[1] Vandereyken, K., Sifrim, A., Thienpont, B. & Voet, T. Methods and applications for single-cell and spatial multi-omics. *Nat. Rev. Genet.* **24**, 494–515 (2023). 36864178 10.1038/s41576-023-00580-2 PMC9979144

[2] Moffitt, J. R., Lundberg, E. & Heyn, H. The emerging landscape of spatial profiling technologies. *Nat. Rev. Genet.* **23**, 741–759 (2022). 35859028 10.1038/s41576-022-00515-3

[3] Strack, R. Highly multiplexed transcriptome imaging. *Nat. Methods* **12**, 486–487 (2015). 26221655 10.1038/nmeth.3426

[4] Angelo, M. et al. Multiplexed ion beam imaging of human breast tumors. *Nat. Med.* **20**, 436–442 (2014). 24584119 10.1038/nm.3488 PMC4110905

[5] Giesen, C. et al. Highly multiplexed imaging of tumor tissues with subcellular resolution by mass cytometry. *Nat. Methods* **11**, 417–422 (2014). 24584193 10.1038/nmeth.2869

[6] Black, S. et al. CODEX multiplexed tissue imaging with DNA-conjugated antibodies. *Nat. Protoc.* **16**, 3802–3835 (2021). 34215862 10.1038/s41596-021-00556-8 PMC8647621

[7] Lin, J. R. et al. Highly multiplexed immunofluorescence imaging of human tissues and tumors using t-CyCIF and conventional optical microscopes. *eLife* **7**, e31657 (2018). 29993362 10.7554/eLife.31657 PMC6075866

[8] Greenbaum, S. et al. A spatially resolved timeline of the human maternal–fetal interface. *Nature* **619**, 595–605 (2023). 37468587 10.1038/s41586-023-06298-9 PMC10356615

[9] Hickey, J. W. et al. Organization of the human intestine at single-cell resolution. *Nature* **619**, 572–584 (2023). 37468586 10.1038/s41586-023-05915-x PMC10356619

[10] de Souza, N., Zhao, S. & Bodenmiller, B. Multiplex protein imaging in tumour biology. *Nat. Rev. Cancer* **24**, 171–191 (2024). 38316945 10.1038/s41568-023-00657-4

[11] Stoltzfus, C. R. et al. CytoMAP: a spatial analysis toolbox reveals features of myeloid cell organization in lymphoid tissues. *Cell Rep.* **31**, 107523 (2020). 32320656 10.1016/j.celrep.2020.107523 PMC7233132

[12] Warchol, S. et al. Visinity: visual spatial neighborhood analysis for multiplexed tissue imaging data. *IEEE Trans. Vis. Comput. Graph.* **29**, 106–116 (2023). 36170403 10.1109/TVCG.2022.3209378 PMC10043053

[13] Schapiro, D. et al. histoCAT: analysis of cell phenotypes and interactions in multiplex image cytometry data. *Nat. Methods* **14**, 873–876 (2017). 28783155 10.1038/nmeth.4391 PMC5617107

[14] Somarakis, A., Van Unen, V., Koning, F., Lelieveldt, B. & Hollt, T. ImaCytE: visual exploration of cellular micro-environments for imaging mass cytometry data. *IEEE Trans. Vis. Comput. Graph.* **27**, 98–110 (2021). 31369380 10.1109/TVCG.2019.2931299

[15] Gut, G., Herrmann, M. D. & Pelkmans, L. Multiplexed protein maps link subcellular organization to cellular states. *Science* **361**, eaar7042 (2018). 30072512 10.1126/science.aar7042

[16] Wahle, P. et al. Multimodal spatiotemporal phenotyping of human retinal organoid development. *Nat. Biotechnol.* **41**, 1765–1775 (2023). 37156914 10.1038/s41587-023-01747-2 PMC10713453

[17] Park, J. et al. Spatial omics technologies at multimodal and single cell/subcellular level. *Genome Biol.* **23**, 256 (2022). 36514162 10.1186/s13059-022-02824-6 PMC9746133

[18] Benjamin, K. et al. Multiscale topology classifies cells in subcellular spatial transcriptomics. *Nature* **630**, 943–949 (2024). 38898271 10.1038/s41586-024-07563-1 PMC11208150

[19] Singh, N. et al. Development of a 2-dimensional atlas of the human kidney with imaging mass cytometry. *JCI Insight* **4**, e129477 (2019). 31217358 10.1172/jci.insight.129477 PMC6629112

[20] Hansen, J. et al. A reference tissue atlas for the human kidney. *Sci. Adv.* **8**, eabn4965 (2022). 35675394 10.1126/sciadv.abn4965 PMC9176741

[21] Raschka, S., Patterson, J. & Nolet, C. Machine learning in Python: main developments and technology trends in data science, machine learning, and artificial intelligence. *Information* **11**, 193 (2020).

[22] Mancini, R., Ritacco, A., Lanciano, G. & Cucinotta, T. XPySom: High-Performance Self-Organizing Maps. In *Proc. 2020 IEEE 32nd International Symposium on Computer Architecture and High Performance Computing (SBAC-PAD)* 209–216 (IEEE, 2020).

[23] Virshup, I. et al. The scverse project provides a computational ecosystem for single-cell omics data analysis. *Nat. Biotechnol.* **41**, 604–606 (2023). 37037904 10.1038/s41587-023-01733-8

[24] Traag, V. A., Waltman, L. & van Eck, N. J. From Louvain to Leiden: guaranteeing well-connected communities. *Sci. Rep.* **9**, 5233 (2019). 30914743 10.1038/s41598-019-41695-z PMC6435756

[25] Kylies, D. et al. Expansion-enhanced super-resolution radial fluctuations enable nanoscale molecular profiling of pathology specimens. *Nat. Nanotechnol.* **18**, 336–342 (2023). 37037895 10.1038/s41565-023-01328-z PMC10115634

[26] Pandit, K. et al. An open source toolkit for repurposing Illumina sequencing systems as versatile fluidics and imaging platforms. *Sci. Rep.* **12**, 5081 (2022). 35332182 10.1038/s41598-022-08740-w PMC8948189

[27] Wong, M. N., Tharaux, P. L., Grahammer, F. & Puelles, V. G. Parietal epithelial cell dysfunction in crescentic glomerulonephritis. *Cell Tissue Res.* **385**, 345–354 (2021). 34453566 10.1007/s00441-021-03513-9 PMC8523405

[28] Grigorieva, I. V. et al. A novel role for GATA3 in mesangial cells in glomerular development and injury. *J. Am. Soc. Nephrol.* **30**, 1641–1658 (2019). 31405951 10.1681/ASN.2018111143 PMC6727249

[29] Stambe, C., Atkins, R. C., Hill, P. A. & Nikolic-Paterson, D. J. Activation and cellular localization of the p38 and JNK MAPK pathways in rat crescentic glomerulonephritis. *Kidney Int.* **64**, 2121–2132 (2003). 14633134 10.1046/j.1523-1755.2003.00324.x

[30] Zimmermann, M. et al. Deep learning-based molecular morphometrics for kidney biopsies. *JCI Insight* **6**, e144779 (2021). 33705360 10.1172/jci.insight.144779 PMC8119189

[31] Kok, H. M., Falke, L. L., Goldschmeding, R. & Nguyen, T. Q. Targeting CTGF, EGF and PDGF pathways to prevent progression of kidney disease. *Nat. Rev. Nephrol.* **10**, 700–711 (2014). 25311535 10.1038/nrneph.2014.184

[32] Ma, F. Y. et al. Blockade of the c-Jun amino terminal kinase prevents crescent formation and halts established anti-GBM glomerulonephritis in the rat. *Lab. Invest.* **89**, 470–484 (2009). 19188913 10.1038/labinvest.2009.2

[33] Ma, F. Y. et al. A pathogenic role for c-Jun amino-terminal kinase signaling in renal fibrosis and tubular cell apoptosis. *J. Am. Soc. Nephrol.* **18**, 472–484 (2007). 17202416 10.1681/ASN.2006060604

[34] Jia, T. et al. The role of platelet-derived growth factor in focal segmental glomerulosclerosis. *J. Am. Soc. Nephrol.* **34**, 241–257 (2023). 36351762 10.1681/ASN.2022040491 PMC10103089

[35] Eymael, J. et al. CD44 is required for the pathogenesis of experimental crescentic glomerulonephritis and collapsing focal segmental glomerulosclerosis. *Kidney Int.* **93**, 626–642 (2018). 29276101 10.1016/j.kint.2017.09.020

[36] Lazareth, H. et al. The tetraspanin CD9 controls migration and proliferation of parietal epithelial cells and glomerular disease progression. *Nat. Commun.* **10**, 3303 (2019). 31341160 10.1038/s41467-019-11013-2 PMC6656772

[37] Foster, L. C. et al. Role of activating protein-1 and high mobility group-I(Y) protein in the induction of *CD44* gene expression by interleukin-1β in vascular smooth muscle cells. *FASEB J.* **14**, 368–378 (2000). 10657993 10.1096/fasebj.14.2.368

[38] DeFronzo, R. A., Reeves, W. B. & Awad, A. S. Pathophysiology of diabetic kidney disease: impact of SGLT2 inhibitors. *Nat. Rev. Nephrol.* **17**, 319–334 (2021). 33547417 10.1038/s41581-021-00393-8

[39] Stringer, C., Wang, T., Michaelos, M. & Pachitariu, M. Cellpose: a generalist algorithm for cellular segmentation. *Nat. Methods* **18**, 100–106 (2021). 33318659 10.1038/s41592-020-01018-x

[40] Tanevski, J., Flores, R. O. R., Gabor, A., Schapiro, D. & Saez-Rodriguez, J. Explainable multiview framework for dissecting spatial relationships from highly multiplexed data. *Genome Biol.* **23**, 97 (2022). 35422018 10.1186/s13059-022-02663-5 PMC9011939

[41] Joodaki, M. et al. Detection of patient-level distances from single cell genomics and pathomics data with optimal transport (PILOT). *Mol. Syst. Biol.* **20**, 57–74 (2024). 38177382 10.1038/s44320-023-00003-8 PMC10883279

[42] Hartung, M. et al. UnPaSt: unsupervised patient stratification by differentially expressed biclusters in omics data. Preprint at 10.48550/arXiv.2408.00200 (2024).

[43] Jensen, L. J. et al. STRING 8—a global view on proteins and their functional interactions in 630 organisms. *Nucleic Acids Res.* **37**, D412–D416 (2009). 18940858 10.1093/nar/gkn760 PMC2686466

[44] Davis, A. P. et al. Comparative Toxicogenomics Database (CTD): update 2023. *Nucleic Acids Res.* **51**, D1257–D1262 (2023). 36169237 10.1093/nar/gkac833 PMC9825590

[45] DeFronzo, R. A. et al. Type 2 diabetes mellitus. *Nat. Rev. Dis. Primers.* **1**, 15019 (2015). 27189025 10.1038/nrdp.2015.19

[46] Schaub, J. A. et al. SGLT2 inhibitors mitigate kidney tubular metabolic and mTORC1 perturbations in youth-onset type 2 diabetes. *J. Clin. Invest.* **133**, e164486 (2023). 36637914 10.1172/JCI164486 PMC9974101

[47] Balzer, M. S. et al. Treatment effects of soluble guanylate cyclase modulation on diabetic kidney disease at single-cell resolution. *Cell Rep. Med.* **4**, 100992 (2023). 37023747 10.1016/j.xcrm.2023.100992 PMC10140477

[48] Hickey, J. W. et al. Spatial mapping of protein composition and tissue organization: a primer for multiplexed antibody-based imaging. *Nat. Methods* **19**, 284–295 (2022). 34811556 10.1038/s41592-021-01316-y PMC9264278

[49] Kuett, L. et al. Three-dimensional imaging mass cytometry for highly multiplexed molecular and cellular mapping of tissues and the tumor microenvironment. *Nat. Cancer* **3**, 122–133 (2022). 35121992 10.1038/s43018-021-00301-w PMC7613779

[50] Wang, X. Q. et al. Spatial predictors of immunotherapy response in triple-negative breast cancer. *Nature* **621**, 868–876 (2023). 37674077 10.1038/s41586-023-06498-3 PMC10533410

[51] Hoch, T. et al. Multiplexed imaging mass cytometry of the chemokine milieus in melanoma characterizes features of the response to immunotherapy. *Sci. Immunol.* **7**, eabk1692 (2022). 35363540 10.1126/sciimmunol.abk1692

[52] Radtke, A. J. et al. IBEX: a versatile multiplex optical imaging approach for deep phenotyping and spatial analysis of cells in complex tissues. *Proc. Natl Acad. Sci. USA* **117**, 33455–33465 (2020). 33376221 10.1073/pnas.2018488117 PMC7776876

[53] Kim, J. et al. Unsupervised discovery of tissue architecture in multiplexed imaging. *Nat. Methods* **19**, 1653–1661 (2022). 36316562 10.1038/s41592-022-01657-2 PMC11102857

[54] Lake, B. B. et al. An atlas of healthy and injured cell states and niches in the human kidney. *Nature.* **619**, 585–594 (2023). 37468583 10.1038/s41586-023-05769-3 PMC10356613

[55] Liu, C. C. et al. Robust phenotyping of highly multiplexed tissue imaging data using pixel-level clustering. *Nat. Commun.* **14**, 4618 (2023). 37528072 10.1038/s41467-023-40068-5 PMC10393943

[56] Schapiro, D. et al. MITI minimum information guidelines for highly multiplexed tissue images. *Nat. Methods* **19**, 262–267 (2022). 35277708 10.1038/s41592-022-01415-4 PMC9009186

[57] Quardokus, E. M. et al. Organ mapping antibody panels: a community resource for standardized multiplexed tissue imaging. *Nat. Methods* **20**, 1174–1178 (2023). 37468619 10.1038/s41592-023-01846-7 PMC10406602

[58] Gnirck, A. C. et al. Endogenous IL-22 is dispensable for experimental glomerulonephritis. *Am. J. Physiol. Renal Physiol.* **316**, F712–F722 (2019). 30724106 10.1152/ajprenal.00303.2018

[59] Flanc, R. S. et al. A pathogenic role for JNK signaling in experimental anti-GBM glomerulonephritis. *Kidney Int.* **72**, 698–708 (2007). 17597698 10.1038/sj.ki.5002404

[60] Walter, K. and Ziesche, F. Apparatus and method, particularly for microscopes and endoscopes, using baseline estimation and half-quadratic minimization for the deblurring of images. European patent WO/2019/185174/A1 (2019).

[61] Ponzetti, M., Chinna Rao Devarapu, G., Rucci, N., Carlone, A. & Saggiomo, V. HistoEnder: a 3D printer-based histological slide autostainer that retains 3D printer functions. *HardwareX* **12**, e00370 (2022). 36345434 10.1016/j.ohx.2022.e00370 PMC9636191

[62] Clausen, B. E. et al. Guidelines for mouse and human DC functional assays. *Eur. J. Immunol.* **53**, e2249925 (2023). 36563126 10.1002/eji.202249925

[63] Wortel, I. M. N. et al. CelltrackR: an R package for fast and flexible analysis of immune cell migration data. *Immunoinformatics* **1**, 100003 (2021). 10.1016/j.immuno.2021.100003 PMC10079262 37034276

[64] Gies, S. E. et al. Optimized protocol for the multiomics processing of cryopreserved human kidney tissue. *Am. J. Physiol. Renal Physiol.* **327**, F822–F844 (2024). 39361723 10.1152/ajprenal.00404.2023

[65] Köster, J. & Rahmann, S. Snakemake—a scalable bioinformatics workflow engine. *Bioinformatics* **28**, 2520–2522 (2012). 22908215 10.1093/bioinformatics/bts480

[66] Kuehl, M., Wong, M. N., Wanner, N., Bonn, S. & Puelles, V. G. Gene count estimation with pytximport enables reproducible analysis of bulk RNA sequencing data in Python. *Bioinformatics* **40**, btae700 (2024). 39565903 10.1093/bioinformatics/btae700 PMC11629965

[67] Andrews, S. FastQC: a quality control tool for high throughput sequence data (2010); http://www.bioinformatics.babraham.ac.uk/projects/fastqc.

[68] Chen, S., Zhou, Y., Chen, Y. & Gu, J. fastp: an ultra-fast all-in-one FASTQ preprocessor. *Bioinformatics* **34**, i884–i890 (2018). 30423086 10.1093/bioinformatics/bty560 PMC6129281

[69] Harrison, P. W. et al. Ensembl 2024. *Nucleic Acids Res.* **52**, D891–D899 (2024). 37953337 10.1093/nar/gkad1049 PMC10767893

[70] Patro, R., Duggal, G., Love, M. I., Irizarry, R. A. & Kingsford, C. Salmon provides fast and bias-aware quantification of transcript expression. *Nat. Methods* **14**, 417–419 (2017). 28263959 10.1038/nmeth.4197 PMC5600148

[71] Muzellec, B., Teleńczuk, M., Cabeli, V. & Andreux, M. PyDESeq2: a Python package for bulk RNA-seq differential expression analysis. *Bioinformatics* **39**, btad547 (2023). 37669147 10.1093/bioinformatics/btad547 PMC10502239

[72] Müller-Dott, S. et al. Expanding the coverage of regulons from high-confidence prior knowledge for accurate estimation of transcription factor activities. *Nucleic Acids Res.* **51**, 10934–10949 (2023). 37843125 10.1093/nar/gkad841 PMC10639077

[73] Badia-I-Mompel, P. et al. decoupleR: ensemble of computational methods to infer biological activities from omics data. *Bioinform. Adv.* **2**, vbac016 (2022). 36699385 10.1093/bioadv/vbac016 PMC9710656

[74] Virshup, I., Rybakov, S., Theis, F. J., Angerer, P. & Wolf, F. A. anndata: Annotated data. *J. Open Source Softw.* **9**, 4371 (2024).

[75] Harris, C. R. et al. Array programming with NumPy. *Nature* **585**, 357–362 (2020). 32939066 10.1038/s41586-020-2649-2 PMC7759461

[76] McKinney, W. pandas: a foundational Python library for data analysis and statistics. *Python High Perform. Sci. Comput.* **14**, 1–9 (2011).

[77] Nishino, R., Unno, Y., Nishino, D, Hido, S. & Loomis, C. Cupy: a numpy-compatible library for nvidia GPU calculations. In *Proc. 31st Confernce on Neural Information Processing Systems* (ed. Braun, M.) 151 (NIPS, 2017).

[78] Pedregosa, F. et al. Scikit-learn: machine learning in Python. *J. Mach. Learn. Res.* **12**, 2825–2830 (2011).

[79] McInnes, L., Healy, J., Saul, N. & Großberger, L. UMAP: uniform manifold approximation and projection. *J. Open Source Softw.* **3**, 861 (2018).

[80] Rey, S. J. & Anselin, L. in *Handbook of Applied Spatial Analysis: Software Tools, Methods and Applications* (eds Fischer, M. M. & Getis, A.) 175–193 (Springer, 2010).

[81] Emons, M. et al. pasta: pattern analysis for spatial omics data. Preprint at https://arxiv.org/abs/2412.01561 (2025).

[82] Hunter, J. D. Matplotlib: a 2D graphics environment. *Comput. Sci. Eng.* **9**, 90–95 (2007).

[83] Waskom, M. L. seaborn: statistical data visualization. *J. Open Source Softw.* **6**, 3021 (2021).

[84] van der Walt, S. et al. scikit-image: image processing in Python. *PeerJ* **2**, e453 (2014). 25024921 10.7717/peerj.453 PMC4081273

[85] Bradski, G. The openCV library. *Dr Dobbs J. Softw. Tools* **25**, 120–123 (2000).

[86] Lowe, D. G. Distinctive image features from scale-invariant keypoints. *Int. J. Comput. Vision* **60**, 91–110 (2004).

[87] Klein, S., Staring, M., Murphy, K., Viergever, M. A. & Pluim, J. P. W. elastix: a toolbox for intensity-based medical image registration. *IEEE Trans. Med. Imaging* **29**, 196–205 (2010). 19923044 10.1109/TMI.2009.2035616

[88] Klein, A. PyElastix—Python wrapper for the Elastix nonrigid registration toolkit. GitHub https://github.com/almarklein/pyelastix (2019).

[89] Polański, K. et al. BBKNN: fast batch alignment of single cell transcriptomes. *Bioinformatics* **36**, 964–965 (2020). 31400197 10.1093/bioinformatics/btz625 PMC9883685

[90] Szklarczyk, D. et al. The STRING database in 2023: protein–protein association networks and functional enrichment analyses for any sequenced genome of interest. *Nucleic Acids Res.* **51**, D638–D646 (2023). 36370105 10.1093/nar/gkac1000 PMC9825434

[91] Charlier, F. et al. Statannotations v.0.6. *Zenodo*10.5281/zenodo.7213391 (2022).

[92] Vaserstein, L. N. Markov processes over denumerable products of spaces, describing large systems of automata. *Probl. Pereda. Inf.* **5**, 64–72 (1969).

[93] Coifman, R. R. & Lafon, S. Diffusion maps. *Appl. Comput. Harmon. Anal.* **21**, 5–30 (2006).

[94] Ellson, J., Gansner, E., Koutsofios, L., North, S. C. & Woodhull, G. in *Graph Drawing* (eds Mutzel, P. et al.) 483–484 (Springer, 2002).

[95] Hagberg, A., Swart, P. & Chult, D. S. Exploring network structure, dynamics, and function using NetworkX. In *Proc. 7th Python in Science Conference* (eds Varoquaux, G. et al.) 11–15 (2008).

[96] Fleming, S. J. et al. Unsupervised removal of systematic background noise from droplet-based single-cell experiments using CellBender. *Nat. Methods* **20**, 1323–1335 (2023). 37550580 10.1038/s41592-023-01943-7

[97] Wolf, F. A., Angerer, P. & Theis, F. J. SCANPY: large-scale single-cell gene expression data analysis. *Genome Biol.* **19**, 15 (2018). 29409532 10.1186/s13059-017-1382-0 PMC5802054

[98] Korsunsky, I. et al. Fast, sensitive and accurate integration of single-cell data with Harmony. *Nat. Methods* **16**, 1289–1296 (2019). 31740819 10.1038/s41592-019-0619-0 PMC6884693

[99] Slowikowski, K., Arevalo, J. & Manning, J. slowkow/harmonypy: harmonypy version 0.0.10. *Zenodo*10.5281/zenodo.12693230 (2024).

[100] Franzén, O., Gan, L.-M. & Björkegren, J. L. M. PanglaoDB: a web server for exploration of mouse and human single-cell RNA sequencing data. *Database J. Biol. Databases Curation* **2019**, baz046 (2019). 10.1093/database/baz046 PMC6450036 30951143

[101] Virtanen, P. et al. SciPy 1.0: fundamental algorithms for scientific computing in Python. *Nat. Methods* **17**, 261–272 (2020). 32015543 10.1038/s41592-019-0686-2 PMC7056644

[102] Skipper, S. & Perktold, J. statsmodels: econometric and statistical modeling with Python. In *Proc. 9th Python in Science Conference* (eds van der Walt, S. & Millman, J.) 92–96 (2010).

[103] Zhu, A., Ibrahim, J. G. & Love, M. I. Heavy-tailed prior distributions for sequence count data: removing the noise and preserving large differences. *Bioinformatics* **35**, 2084–2092 (2019). 30395178 10.1093/bioinformatics/bty895 PMC6581436

[104] Okabayashi, Y., Kuehl, M. B. & Puelles, V. Pathology-oriented multiplexing enables integrative disease mapping—imaging files. *Zenodo*10.5281/zenodo.15212140 (2025). 10.1038/s41586-025-09225-2 PMC12350167 40681898

[105] Kuehl, M. B., Okabayashi, Y. & Puelles, V. Pathology-oriented multiplexing enablesintegrative disease mapping—processing code and 3D printing files. *Zenodo*10.5281/zenodo.15211354 (2025).

