# Supplementary material for: Pathology-oriented multiplexing enables integrative disease mapping
Source: Nature. 2025 Jul 18;644(8076):516–26. doi: 10.1038/s41586-025-09225-2 (PMC12350167; doi:10.1038/s41586-025-09225-2)
Supplement: Supplementary file 2 — Reporting Summary [file 41586_2025_9225_MOESM2_ESM.pdf]

nature portfolio

Corresponding author(s): Victor Puelles

Last updated by author(s): May 6, 2025

Reporting Summary

Nature Portfolio wishes to improve the reproducibility of the work that we publish. This form provides structure for consistency and transparency in reporting. For further information on Nature Portfolio policies, see our Editorial Policies and the Editorial Policy Checklist.

Please do not complete any field with "not applicable" or n/a. Refer to the help text for what text to use if an item is not relevant to your study.
For final submission: please carefully check your responses for accuracy; you will not be able to make changes later.

Statistics

For all statistical analyses, confirm that the following items are present in the figure legend, table legend, main text, or Methods section.

n/a	Confirmed	
☐	☒	The exact sample size (*n*) for each experimental group/condition, given as a discrete number and unit of measurement
☐	☒	A statement on whether measurements were taken from distinct samples or whether the same sample was measured repeatedly
☐	☒	The statistical test(s) used AND whether they are one- or two-sided<br>*Only common tests should be described solely by name; describe more complex techniques in the Methods section.*
☐	☒	A description of all covariates tested
☐	☒	A description of any assumptions or corrections, such as tests of normality and adjustment for multiple comparisons
☐	☒	A full description of the statistical parameters including central tendency (e.g. means) or other basic estimates (e.g. regression coefficient) AND variation (e.g. standard deviation) or associated estimates of uncertainty (e.g. confidence intervals)
☐	☒	For null hypothesis testing, the test statistic (e.g. *F*, *t*, *r*) with confidence intervals, effect sizes, degrees of freedom and *P* value noted<br>*Give P values as exact values whenever suitable.*
☒	☐	For Bayesian analysis, information on the choice of priors and Markov chain Monte Carlo settings
☒	☐	For hierarchical and complex designs, identification of the appropriate level for tests and full reporting of outcomes
☐	☒	Estimates of effect sizes (e.g. Cohen's *d*, Pearson's *r*), indicating how they were calculated

Our web collection on statistics for biologists contains articles on many of the points above.

Software and code

Policy information about availability of computer code

Data collection	Data collection was performed using: Leica Application Suite X software (Ver3.7.6, Leica Microsystems), ZEN2.6 (Zeiss) and ZEN 3.5 System (Zeiss), TrackMate plugin from Fiji® (v7.10.2), CelltrackR package, and Fluidigm CyTOF software v.01,
Data analysis	For general statistical analysis, we used GraphPad Prism (V10.4.2). The code for the 3D printer-based liquid handling system and the STL files for 3D printing, the spatiomic library code and the code for the analysis of all datasets are available through Zenodo with DOI: https://doi.org/10.5281/zenodo.15211354. The latest development version of spatiomic can be accessed at: https://github.com/complextissue/spatiomic. The documentation for spatiomic is available at: https://spatiomic.complextissue.com and includes example workflows for common analyses, including code to download example data to run a full example. The bulk RNA-sequencing workflow can be accessed at: https://github.com/complextissue/snakemake-bulk-rna-seq-workflow/.<br>MistyR is available at https://github.com/saezlab/mistyR/. PILOT is available at https://github.com/CostaLab/PILOT/. UnPaSt is available at https://github.com/ozolotareva/UnPaSt. The custom XPySOM adaptation that includes support for the Pearson correlation distance metric is available at: https://github.com/complextissue/xpysom.

For manuscripts utilizing custom algorithms or software that are central to the research but not yet described in published literature, software must be made available to editors and reviewers. We strongly encourage code deposition in a community repository (e.g. GitHub). See the Nature Portfolio guidelines for submitting code & software for further information.

Data

Policy information about availability of data

All manuscripts must include a data availability statement. This statement should provide the following information, where applicable:

- Accession codes, unique identifiers, or web links for publicly available datasets
- A description of any restrictions on data availability
- For clinical datasets or third party data, please ensure that the statement adheres to our policy

The bulk RNA-sequencing data from NTS-treated mice have been deposited into the Hamburg University Research Data Repository with https://doi.org/10.25592/uhhfdm.17394. The public single cell and single nucleus RNA-sequencing datasets used in this study are available through Gene Expression Omnibus with accessions GSE220939 and GSE209821. PathoPlex animal multiplexed imaging data is available through Zenodo with DOI: https://doi.org/10.5281/zenodo.15212140. In addition, Source data are provided with this paper.
For human data, as a patient re-identification key is retained internally for scientific continuity of ongoing projects, and historical versions of data containing patient identifiers persist in secure institutional servers and physical laboratory records, the raw microscopy data cannot be fully anonymized and therefore cannot be deposited in a public repository in accordance with General Data Protection Regulation (GDPR). Raw data can be made available upon reasonable request and subject to a material and data user agreement (MDUA) that ensures appropriate safeguards for data protection and privacy in compliance with GDPR. The senior corresponding author will respond to data requests, aiming to answer within 72hrs, and provide data up to one month after the MDUA has been signed by both parties.

Research involving human participants, their data, or biological material

Policy information about studies with human participants or human data. See also policy information about sex, gender (identity/presentation), and sexual orientation and race, ethnicity and racism.

Reporting on sex and gender	Given the limited sample size, we did not take into account sex as selection criteria or as part of our analysis.
Reporting on race, ethnicity, or other socially relevant groupings	Parts of our study were conducted on kidney biopsies from three cohorts (France, Germany and USA). This was not considered in the analysis.
Population characteristics	As our experiments were performed in archival tissues, we did not control population characteristics. We report age in one of of clinical cohorts as it may represent a confounding factor. However, we do not correct or adjust for it given our limited sample size.
Recruitment	The first study used specimens from patients who presented with renal impairment and/or proteinuria and were diagnosed with diabetic kidney disease after biopsy was performed. As a control group, we used the noncancerous portion of kidney tissue from patients who had undergone nephrectomy for renal cell carcinoma.<br>The second study included research biopsies from 3 groups of subjects: (1) healthy controls, (2) patients with type 2 diabetes that received SGLT2 inhibitors, and (3) patients with type 2 diabetes that did not receive SGLT2 inhibitors. Patients volunteered for a kidney biopsy as there was no clinical indication.
Ethics oversight	Ethical approvals were obtained from the Institutional Review Board (IRB) of the RWTH Aachen University Medical Center (EK-016/17), the local Ethics Committees of the Chamber of Physicians in Hamburg (PV4806) and Freiburg (Ethikvotum 10008/09), the Paris Ethics Committee (IRB00003888, FWA00005831), and the Colorado Ethics Committee (NCT03584217 and NCT03620773). All tissue collections were performed in accordance with the ethical principles stated by the Declaration of Helsinki.

Note that full information on the approval of the study protocol must also be provided in the manuscript.

Field-specific reporting

Please select the one below that is the best fit for your research. If you are not sure, read the appropriate sections before making your selection.

☒ Life sciences ☐ Behavioural & social sciences ☐ Ecological, evolutionary & environmental sciences

For a reference copy of the document with all sections, see nature.com/documents/nr-reporting-summary-flat.pdf

Life sciences study design

All studies must disclose on these points even when the disclosure is negative.

Sample size	This study reports a new technology and applies it to 3 different case studies, one in an experimental model of immune-mediated kidney disease, one in a clinical cohort of patients with advanced diabetic kidney disease, and one in a research cohort of patients with early type 2 diabetes. No statistical method were used to determine sample size as they were chosen based on availability of archival biopsy material (n=18-20 per group in clinical biopsies and 5-7 in research biopsies). Previous experience for experimental immune-mediated kidney disease (at least n=3 for each experimental group): PMID: 33622974 , PMID: 32446933 and PMID: 40050432
Data exclusions	Stained images in which the primary antibody did not pass quality controls (e.g. comparison to previous literature or antibody validation

Data exclusions	steps) or images containing artifacts (e.g. tissue that detached from the glass) were excluded from the analysis.
Replication	During the developmental phase, experiments were repeated and successfully reproduced at least 3 times, this includes short multiplexed imaging experiments for proof-of-principle, quality control tests, 3D printing, coating agents and repositioning workflows. After the method and steps were established, we proceeded to experimental cases. All multiplex imaging experiments (Figures 2, 3 and 4) were performed on a minimum of 3 samples per experimental group. For Figure 2, a total of 10 mice were analyzed. For Figure 3, a total of 38 patients and for Figure 4 a total of 18 patients. Negative controls were performed at least once for each sample used, including cycles with secondaries only. Additional quality control steps are extensively discussed in the methodology section. Every attempt to replicate the data has been successful.
Randomization	We worked with archival tissue specimens to showcase the development and application of a new technology. No randomization was conducted as we had no impact on tissue collection and allocation.
Blinding	When possible, blinding was used. For example, when analyzing early phase of crescentic glomerulonephritis, as structures did not appear pathological or in the characterization of the in vivo effectiveness of the JNK inhibitor (where lesions were not evident). When pathological lesions were present, then blinding was not possible as pathological groups would become evident. For human studies, all groups were included in imaging chambers, so all steps were performed for all groups in parallel and under identical conditions. As we needed to correlate with clinical parameters, blinding was not possible.

Reporting for specific materials, systems and methods

We require information from authors about some types of materials, experimental systems and methods used in many studies. Here, indicate whether each material, system or method listed is relevant to your study. If you are not sure if a list item applies to your research, read the appropriate section before selecting a response.

Materials & experimental systems

n/a	Involved in the study	
[ ]	[X]	Antibodies
[ ]	[X]	Eukaryotic cell lines
[X]	[ ]	Palaeontology and archaeology
[ ]	[X]	Animals and other organisms
[X]	[ ]	Clinical data
[X]	[ ]	Dual use research of concern
[X]	[ ]	Plants

Methods

n/a	Involved in the study	
[X]	[ ]	ChIP-seq
[X]	[ ]	Flow cytometry
[X]	[ ]	MRI-based neuroimaging

Antibodies

Antibodies used	Primary antibodies and lectins<br>For human samples. ABCG2 (Santa Cruz; sc-377176, 1:200), ACE-2 (R&D Systems; AF933, 1:200), Adiponectin (Thermo Fisher Scientific; MA1-054, 1:200), AIF (Cell Signaling Technology; 5318, 1:200), AKAP12 (Proteintech; 25199-1-AP, 1:600), AKR1B1 (Thermo Fisher Scientific; PA5-82915, 1:500), AKR1C1 (Thermo Fisher Scientific; MA5-32842, 1:200), Alpha B Crystallin (Proteintech; 68001-1-Ig, 1:1000), ANXA3 (Sigma-Aldrich; HPA013398, 1:200), αSMA-FITC conjugate (Sigma-Aldrich; F3777, 1:800), Aquaporin 2 (Alomone Labs; AQP-002, 1:400), β-Actin (Sigma-Aldrich; A5441, 1:1500), β-Catenin (Abcam; ab6302, 1:2000), β-Tubulin (Cell Signaling Technology; 2128, 1:150), Calbindin-D (Sigma-Aldrich; C9848, 1:3000), Calpain small subunit 1 (Abcam; ab92333, 1:200), Calpastatin (Abcam; ab244460, 1:200), Calreticulin (Abcam; ab92516, 1:300), Carbonic Anhydrase IX (R&D Systems; AF2188, 1:50), Catalase (Proteintech; 66765-1-Ig, 1:300), CD3 (Abcam; ab11089, 1:200), CD4 (R&D Systems; AF-379-NA, 1:100), CD8 (Agilent; M710301-2, 1:200), CD34 (Agilent; GA63261-2, 1:50), CD41 (Thermo Fisher Scientific; PA5-79526, 1:500), CD42b (Abcam; ab227669, 1:100), CD44 (Cell Signaling Technology; 5640S, 1:200), CD44-Alexa Fluor 647 conjugate (Bio Legend; 103018, 1:200), CD68 (Bio Legend; 916104, 1:200), CD79α (Agilent; M705001-2, 1:200), CD200 (R&D Systems; AF2724, 1:100), CD206 (Proteintech; 60143-1-Ig, 1:2000), c-Fos (Abcam; ab190289, 1:600), Claudin 1 (Abcam; ab15098, 1:500), Claudin 10 (Thermo Fisher Scientific; 38-8400, 1:100), Collagen I (Southern Biotech; 1310-01, 1:200), Collagen III (Abcam; ab7778, 1:200), Collagen IV (Abcam; ab6586, 1:200), Collagen V (Abcam; ab7046, 1:100), Cubilin (R&D Systems; AF3700, 1:200), Cyclin B1 (Cell Signaling Technology; 12231, 1:100), Cytochrome C (Abcam; ab110325, 1:200), Cytokeratin 7 (Agilent; GA61961-2, 1:300), Cytokeratin 8 (R&D Systems; MAB3165-SP, 1:300), Cytokeratin 19 (Abcam; ab52625, 1:300), C1QA (Proteintech; 67063-1-Ig, 1:1000), DACH1 (Sigma-Aldrich; HPA012672, 1:200), Decorin (R&D Systems; AF143, 1:50), E-Cadherin (R&D Systems; AF648, 1:200), EEA1 (Santa Cruz; sc-137130, 1:100), EHD3 (LSBio; LS-C133741, 1:150), Endomucin (Sigma-Aldrich; HPA005928, 1:100), eNOS (Abcam; ab76198, 1:200), Ezrin (Cell Signaling Technology; 3145S, 1:300), FAM189A2 (Thermo Fisher Scientific; PA5-63414, 1:200), Fibronectin (Abcam; ab2413, 1:200), FKBP51 (R&D Systems; AF4094-SP, 1:50), FXYD4 (Thermo Fisher Scientific; PA5-63570, 1:200), GFAP (Thermo Fisher Scientific; 14-9892-82, 1:200), Glucocorticoid Receptor (Cell Signaling Technology; 3660, 1:2000), Glutathione Peroxidase 1 (R&D Systems; AF3798, 1:100), Glutathione Peroxidase 3 (R&D Systems; AF4199, 1:50), Glycophorin A (R&D Systems; MAB1228-SP, 1:500), GRP78 (Proteintech; 11587-1-AP, 1:200), HB-EGF (R&D Systems; AF-259, 1:100), Histone H3 (Cell Signaling Technology; 4499, 1:400), HMOX1 (Thermo Fisher Scientific; MA1-112, 1:200), HSD11B2 (R&D Systems; MAB8630-SP, 1:100), KIM-1 (R&D Systems; AF1750, 1:200), IBA1 (Thermo Fisher Scientific; MA5-27726, 1:500), IDH1 R132H (Dianova; DIA-H09, 1:200), IL-1RA (Abcam; ab124962, 1:200 – specificity issues were raised by the provider after our experiments were completed. We have kept it in the panel as none of our findings were affected and we did not perform any biological inferences based on this antibody), iNOS (Thermo Fisher Scientific; MA5-41652, 1:200), Integrin-α1 (R&D Systems; AF5676, 1:300), Integrin-α3 (Proteintech; 66070-1-Ig, 1:2000), Integrin-β1 (Abcam; ab179471, 1:800), Ki-67 (Agilent; M724029-2, 1:200), Laminin (Abcam; ab11575, 1:200), LAMP1 (Cell Signaling Technology; 9091, 1:300), LC3B (Cell Signaling Technology; 3868, 1:300), LEL-DyLight 649 conjugate (Vector Laboratories; DL-1178, 1:300), LTL biotinylated (Vector Laboratories; B-1325-2, 1:500), MCT1 (Thermo Fisher Scientific; MA5-18288, 1:300), MerTK (R&D Systems; AF591, 1:200), MPO (R&D Systems; MAB3174, 1:200), Nephrin (Progen; GP-N2, 1:150), Neurofilament (Agilent; IR607, 1:200), Nox4 (R&D Systems; MAB8158,

1:300), NQO1 (Proteintech; 67240-1-Ig, 1:2500), OLIG2 (Bio SB; BSB 2561, 1:200), p62 (Cell Signaling Technology; 39749, 1:400), PCK1 (Proteintech; 66862-1-Ig, 1:400), PCNA (Abcam; ab29, 1:2000), PDGFRβ (Cell Signaling Technology; 3169, 1:100), PDI (Cell Signaling Technology; 45596S, 1:400), Periostin (R&D Systems; AF3548, 1:150), Phospho-AMPKα (Cell Signaling Technology; 2535, 1:200), Phospho-c-Jun (Abcam; ab32385, 1:200), Phospho-Erk1/2 (Cell Signaling Technology; 4370, 1:250), Phospho-Ezrin/Radixin/Moesin (Cell Signaling Technology; 3726, 1:200), Phospho-GSK-3β (Cell Signaling Technology; 9323, 1:100), Phospho-Histone H3 (Cell Signaling Technology; 9701, 1:200), Phospho-JAK2 (Thermo Fisher Scientific; MA5-42424, 1:100), Phospho-S6 Ribosomal Protein (Cell Signaling Technology; 4858S, 1:300), Phospho-SMAD2 (Thermo Fisher Scientific; 44-244G, 1:200), Phospho-SMAD3 (Thermo Fisher Scientific; PA5-104940, 1:200), Phospho-STAT1 (Cell Signaling Technology; 9167S, 1:400), Phospho-STAT3 (Abcam; ab76315, 1:200), PITX2 (R&D Systems; AF7388, 1:100), Podocin (Sigma-Aldrich; P0372, 1:3000), Proteasome 20S LMP7 (Abcam; ab3329, 1:400), Rab5A (Cell Signaling Technology; 46449, 1:300), RAB7 (Abcam; ab137029, 1:200), RAP1GAP (Abcam; ab244259, 1:300), RCAS1 (Cell Signaling Technology; 12290, 1:200), Sclerostin (Thermo Fisher Scientific; PA5-37943, 1:100), SirT1 (Cell Signaling Technology; 8469, 1:200), SLC12A3 (Thermo Fisher Scientific; MA5-41643, 1:200), SOD1 (Proteintech; 67480-1-Ig, 1:400), SOD2 (Thermo Fisher Scientific; PA5-30604, 1:300), SRB1 (Abcam; ab217318, 1:300), STAT2 (R&D Systems; MAB16661, 1:200), Survivin (Cell Signaling Technology; 2808, 1:300), Talin 1 (Abcam; ab71333, 1:200), TRPC6 (Abcam; ab233413, 1:200), Ubiquityl-Histone H2B (Cell Signaling Technology; 5546T, 1:200), Uromodulin (R&D Systems; AF5144, 1:300), Villin 1 (Abcam; ab52102, 1:200), Vimentin (Progen; GP53, 1:200), von Willebrand Factor (Agilent; A008229-2, 1:200), WT1 (Agilent; IS05530-2, 1:200), ZO-1 (Thermo Fisher Scientific; 61-7300, 1:250).
For mouse samples. ACE-2 (R&D Systems; AF933, 1:200), AIF (Cell Signaling Technology; 5318, 1:200), AKAP12 (Proteintech; 25199-1-AP, 1:600), ANXA3 (Sigma-Aldrich; HPA013398, 1:200), αSMA-FITC conjugate (Abcam; F3777, 1:800), Aquaporin 2 (Alomone labs; AQP-002, 1:400), Calreticulin (Abcam; ab92516, 1:300), Caspase 1 p20 (Thermo Fisher Scientific; PA5-99390, 1:200), CD3 (Abcam; ab1108, 1:200), CD4 (Abcam; ab183685, 1:200), CD41 (Thermo Fisher Scientific; PA5-79526, 1:500), CD42b (Abcam; ab227669, 1:100), CD44-Alexa Fluor 647 conjugate (Bio Legend; 103018, 1:200), CD45 (Cell Signaling Technology; 70257, 1:200), c-Fos (Abcam; ab190289, 1:600), Collagen I (Southern Biotech; 1310-01, 1:200), Collagen IV (Abcam; ab6586, 1:200), Cytochrome C (Abcam; ab110325, 1:200), DACH1 (Sigma-Aldrich; HPA012672, 1:200), E-Cadherin (R&D Systems; AF648, 1:200), Endomucin (Santa Cruz; sc-65495, 1:200), Fibronectin (Abcam; ab2413, 1:200), Histone H3 (Cell Signaling Technology; 4499, 1:400), IBA1 (Thermo Fisher Scientific; MA5-27726, 1:500), IL-1RA (Abcam; ab124962, 1:200 – specificity issues were raised by the provider after our experiments were completed. We have kept it in the panel as none of our findings were affected and we did not perform any biological inferences based on this antibody), Ki67 (Abcam; ab15580, 1:200), Lamin B1 (Santa Cruz; sc-374015, 1:200), Laminin (Abcam; ab11575, 1:200), LTL biotinylated (Vector Laboratories; B-1325-2, 1:500), Nephrin (Progen; GP-N2, 1:150), PCNA (Abcam; ab29, 1:2000), PDI (Cell Signaling Technology; 45596S, 1:400), Phospho-Ezrin/Radixin/Moesin (Cell Signaling Technology; 3726, 1:200), Podocin (Sigma-Aldrich; P0372, 1:3000), Podoplanin (R&D Systems; AF3244-SP, 1:200), Synaptopodin (Synaptic Systems; 163 004, 1:200), Tyrosine Hydroxylase (Cell Signaling Technology; 45648, 1:200), Ubiquityl-Histone H2B (Cell Signaling Technology; 5546T, 1:200), β-Actin (Sigma-Aldrich; A5441, 1:1500), Vimentin (Progen; GP53, 1:200), von Willebrand Factor (Agilent; A008229-2, 1:200).

Secondary antibodies and biotin-binding proteins
Secondary antibodies were diluted in a ratio ranging from 1:200 to 1:300. Antibodies: goat anti-guinea pig IgG Alexa Fluor 488 (Thermo Fisher Scientific; A-11073), goat anti-guinea pig IgG Alexa Fluor 555 (Thermo Fisher Scientific; A-21435), donkey anti-mouse IgG Alexa Fluor 488 (Thermo Fisher Scientific; A-21202), donkey anti-mouse IgG Alexa Fluor 555 (Thermo Fisher Scientific; A-31570), donkey anti-mouse IgG Alexa Fluor 647 (Thermo Fisher Scientific; A-31571), donkey anti-rabbit IgG Alexa Fluor 488 (Thermo Fisher Scientific; A-21206), donkey anti-rabbit IgG Alexa Fluor 555 (Thermo Fisher Scientific; A-31572), donkey anti-rabbit IgG Alexa Fluor 647 (Thermo Fisher Scientific; A-31573), donkey anti-goat IgG Alexa Fluor 488 (Thermo Fisher Scientific; A-11055), donkey anti-goat IgG Alexa Fluor 555 (Thermo Fisher Scientific; A-21432), donkey anti-rat IgG Alexa Fluor 488 (Thermo Fisher Scientific; A-21208), donkey anti-rat IgG Alexa Fluor 555 (Thermo Fisher Scientific; A78945), donkey anti-sheep IgG Alexa Fluor 488 (Thermo Fisher Scientific; A-11015), donkey anti-sheep IgG Alexa Fluor 555 (Thermo Fisher Scientific; A-21436), streptavidin Alexa Fluor 488 (Thermo Fisher Scientific; S11223), streptavidin Alexa Fluor 555 (Thermo Fisher Scientific; S21381).

Validation

To ensure that antibodies were validated and their staining quality was reliable, we relied on multiple levels of evidence. These include vendor specifications (i.e. recommended concentrations), the absence of primary antibody after elution steps, established staining patterns for every included antibody in the scientific literature, and references to available data from the Human Protein Atlas. These quality control steps were performed by at least 3 different team members. This process was repeated for every antibody included in this study. We provide confirmation of each staining pattern for every antibody in Supplementary Data 1 and 2.

Eukaryotic cell lines

Policy information about cell lines and Sex and Gender in Research

Cell line source(s)

The parietal epithelial cell (PEC) line was provided by A/Prof. Olivia Lenoir and Prof. Pierre-Louis Tharaux

Authentication

In order to preserve their identity, all cell lines were maintained at low passages. This practice ensures consistency in the characteristics and behavior of the cells.

Mycoplasma contamination

It was confirmed that all cell lines were free of any contamination of mycoplasma.

Commonly misidentified lines
(See ICLAC register)

No commonly misidentified cell lines were used in this study.

Animals and other research organisms

Policy information about studies involving animals; ARRIVE guidelines recommended for reporting animal research, and Sex and Gender in Research

Laboratory animals

All experimental animals were housed at ambient temperature of 20±2 °C, humidity of 55±10% and a light/dark cycle of 12 h/12h. Archival tissues from 8-12 week old C57BL/6J mice were used for the experimental glomerulonephritis experiment.

10-12 week-old Sprague Dawley rats were used for prevention experiments with JNK inhibitors, and Wister Kyoto (WKY) rats were used for therapeutic experiments. 12 and 24-week old BTBR-Lepob/ob (BTBRob/ob) mice were used as DKD model.

Wild animals	This study did not involve any wild animals.
Reporting on sex	All studies were performed in male rodents as kidney disease is more severe in males and our aim was to maximize pathological effects and potential interventions.
Field-collected samples	This study did not involve any samples collected from the field.
Ethics oversight	All animal experimental protocols were approved by the respective IRB in Hamburg, Melbourne (N047/20 and MMCB/2006/29), Paris (358-86/609EEC) and Heidelberg (H2052-2071/23)

Note that full information on the approval of the study protocol must also be provided in the manuscript.

Plants

Seed stocks	Not applicable
Novel plant genotypes	Not applicable
Authentication	Not applicable

This checklist template is licensed under a Creative Commons Attribution 4.0 International License, which permits use, sharing, adaptation, distribution and reproduction in any medium or format, as long as you give appropriate credit to the original author(s) and the source, provide a link to the Creative Commons license, and indicate if changes were made. The images or other third party material in this article are included in the article's Creative Commons license, unless indicated otherwise in a credit line to the material. If material is not included in the article's Creative Commons license and your intended use is not permitted by statutory regulation or exceeds the permitted use, you will need to obtain permission directly from the copyright holder. To view a copy of this license, visit http://creativecommons.org/licenses/by/4.0/
