# Supplementary material for: Pathology-oriented multiplexing enables integrative disease mapping
Source: Nature. 2025 Jul 18;644(8076):516–26. doi: 10.1038/s41586-025-09225-2 (PMC12350167; doi:10.1038/s41586-025-09225-2)
Supplement: Supplementary file 1 — Supplementary Figs. 1–12, Supplementary Data 1 and 2 and Supplementary Video legend. [file 41586_2025_9225_MOESM1_ESM.docx]

**Supplementary Figure 1:** **Current state-of-the-art of multiplexed imaging.**

(a) Graphical representation of current available methods for multiplexed imaging based on image resolution (per pixel) and number of targets reported. (b) Classical imaging mass cytometry (resolution: 1000 nm per pixel) in a normal human kidney. (c) Confocal microscopy (resolution: approx. 80 nm per pixel) in a normal mouse kidney. White arrow shows nucleoli. Scale bars represent 40 μm.

**Supplementary Figure 2: Proposal for the development of next-generation multiplexed imaging.** There is a need to reach integrative tissue mapping that combines multiple layers, and this can be best achieved with computational strategies that merge these.

**Supplementary Figure 3. Application of PathoPlex in pathological human tissues.**

Exemplary images showcasing different protein targets in different pathological human organs, including liver (autoimmune hepatitis, AIH), brain (meningioma), and kidney (focal segmental glomerulosclerosis, FSGS). Scale bar: 50 μm. vWF, von Willebrand factor; SRB1, scavenger receptor class B member 1; u-H2B, ubiquityl-histone h2b; PDI, protein disulfide isomerase; EZR, ezrin; p-rp-S6, phospho-S6 ribosomal protein; p-H3, phospho-histone-H3; PCNA, proliferating cell nuclear antigen; COL4, collagen type IV; LAMP1, lysosomal-associated membrane protein 1; VMT, vimentin; FN, fibronectin; WT1, Wilms tumor 1; ACE2, angiotensin-converting enzyme 2;  CK19, cytokeratin 19; ANXA3, annexin A3; LTL, lotus tetragonolobus lectin. Scale bars: 100 μm.

**Supplementary Figure 4: Application of PathoPlex in normal human tissues**

Exemplary images showcasing different protein targets in different normal human organs, including liver, brain, and kidney. Scale bar: 50 μm. vWF, von Willebrand factor; SRB1, scavenger receptor class B member 1; u-H2B, ubiquityl-histone h2b; PDI, protein disulfide isomerase; EZR, ezrin; p-rp-S6, phospho-S6 ribosomal protein; p-H3, phospho-histone-H3; PCNA, proliferating cell nuclear antigen; COL4, collagen type IV; LAMP1, lysosomal-associated membrane protein 1; VMT, vimentin; FN, fibronectin; WT1, Wilms tumor 1; ACE2, angiotensin-converting enzyme 2;  CK19, cytokeratin 19; ANXA3, annexin A3; LTL, lotus tetragonolobus lectin. Scale bars: 100 μm.

**Supplementary Figure 5: Temporal stability of signal intensity across imaging cycles – part 1**

Temporal stability of signal intensity (99.95th percentile) across imaging cycles. Line plots showing the evolution of the 99.95th percentile (orange) for each acquisition wavelength over sequential imaging cycles. Secondary-only cycles (blue) show consistently low 99.95th percentile intensities with no discernible trend of signal increase, indicating no carryover or non-specific accumulation.

**Supplementary Figure 6: Temporal stability of signal intensity across imaging cycles – part 2**

Temporal stability of signal intensity (mean) across imaging cycles. Line plots showing the evolution of the mean intensity (orange) for each acquisition wavelength over sequential imaging cycles. Secondary-only cycles (blue) show consistently low mean intensities with no discernible trend of signal increase, indicating no carryover or non-specific accumulation.

**Supplementary Figure 7: Evaluation of restaining fidelity over extended imaging cycles**

(a) Representative images of staining (cycle 9) and restaining (cycle 70) of the same marker at early and late imaging cycles and their respective histograms, indicating preserved epitope accessibility. Histograms illustrate the pixel intensity distribution from the original staining (blue) and restaining (orange) for each marker, demonstrating signal consistency over 61 cycles. Differences in intensity appear to be due to variable antibody concentration and binding. To allow delineation of high intensity (specific) signal, pixel counts are shown on a Log_10_ scaled axis. Scale bar: 20 μm.

**Supplementary Figure 8: Performance of reference labels for image alignment**

(a) Exemplary images showing DAPI, DRAQ5 and NHS-E over 25 cycles. (b) Structural Similarity Index Metrics (SSIM) and Mean Squared Error (MSE) show equal performance of each referencing method, even DAPI that loses specificity for the nuclear compartment allows accurate referencing of multiplexing imaging rounds. NS: not statistically significant (Kruskal-Wallis test). Scale bar: 50 μm.

a

b

**Supplementary Figure 9: Customizable chambers and liquid handling**

(a) Dimensions of the NEXTERION coverslip (at least 40 samples). As this coverslip needs to be placed on a microscopy system, a chamber frame can be 3D-printed. (b) A 3D printer can also be used as a liquid handling system, including addition, incubation and removal of solutions. (c) Hands-on user time is significantly reduced using a 3D printer-based automated liquid handling system.

a

b

c

**Supplementary Figure 10: Integrated cell segmentation workflow**

(a) Nuclear segmentations based on DRAQ5™ and CellPose^39^ were radially dilated. (b) Cell counts per image showcased no significant changes between disease conditions or imaging plates. (c) Cell communities were identified by averaging the abundances of pixel-based clusters in each cell area and subsequent Leiden graph clustering was performed. (d) Detected cell communities exhibited disease specificity. Scale bar: 100 μm

a

b

c

d

**Supplementary Figure 11: Comparison between 1 hour and overnight incubation of primary antibodies**

(a) Representative images of murine kidney tissue stained using 1 hour (1h) or overnight incubation steps. Images are shown with intensities clipped to 0 - 4000 for consistent comparison. Their respective histograms show the distribution of pixel intensities for each marker across five independent images with a similar overall shape, indicating that the 1h staining provides a robust signal compared to the overnight protocol. X- and y-axis are Log10 scaled. (b) PathoPlex performed with 1-hour incubation. 12 markers were acquired across eight imaging cycles. For visualization, channel intensity histograms were clipped to the 50th - 99.5th percentile range and normalized to the full 16-bit dynamic range. Scale bar: 50 μm

a

b

**Supplementary Figure 12: The effect of marker/cycle order on pixel-based cluster abundance**

Scatter plot showing the Log_2_ fold change (FC) of regulated clusters (as identified in the combined analysis) in each individual plate used for the multiplexed imaging experiment in Figure 3. Each point represents a specific cluster, with red points representing plate 1 and blue points representing plate 2. The x-axis represents the Log_2_ FC relative to healthy controls. Clusters are ordered according to their rank in the combined analysis. The directional consistency of Log_2_ FC across plates, despite small absolute differences, confirms the reliability of the observed regulatory patterns with minimal impact of marker order.

**Supplementary Data 1: Validation of every included antibody for Figure 2**

**Supplementary Data 2: Validation of every included antibody for Figures 3 and 4**

Continues next page

Continues next page

**Supplementary Video: 3D printing-based liquid handling solution**

This video shows the use of a 3D printer with custom 3D-printed cartridges and imaging chambers to perform

automated liquid handling following the PathoPlex protocol.
